# Rheological Characterization and Theoretical Modeling Establish Molecular Design Rules for Tailored Dynamically Associating Polymers

**DOI:** 10.1021/acscentsci.2c00432

**Published:** 2022-09-12

**Authors:** Pamela C. Cai, Bo Su, Lei Zou, Matthew J. Webber, Sarah C. Heilshorn, Andrew J. Spakowitz

**Affiliations:** † Department of Chemical Engineering, Stanford University, Stanford, California 94305, United States; ‡ Department of Chemical & Biomolecular Engineering, University of Notre Dame, Notre Dame, Indiana 46556, United States; § Department of Materials Science and Engineering, Stanford University, Stanford, California 94305, United States

## Abstract

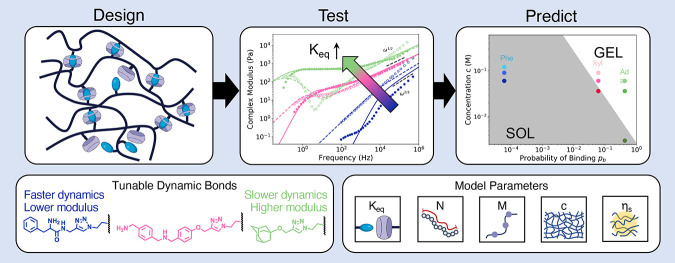

Dynamically associating polymers have long been of interest due to their highly tunable viscoelastic behavior. Many applications leverage this tunability to create materials that have specific rheological properties, but designing such materials is an arduous, iterative process. Current models for dynamically associating polymers are phenomenological, assuming a structure for the relationship between association kinetics and network relaxation. We present the Brachiation model, a molecular-level theory of a polymer network with dynamic associations that is rooted in experimentally controllable design parameters, replacing the iterative experimental process with a predictive model for how experimental modifications to the polymer will impact rheological behavior. We synthesize hyaluronic acid chains modified with supramolecular host–guest motifs to serve as a prototypical dynamic network exhibiting tunable physical properties through control of polymer concentration and association rates. We use dynamic light scattering microrheology to measure the linear viscoelasticity of these polymers across six decades in frequency and fit our theory parameters to the measured data. The parameters are then altered by a magnitude corresponding to changes made to the experimental parameters and used to obtain new rheological predictions that match the experimental results well, demonstrating the ability for this theory to inform the design process of dynamically associating polymeric materials.

## Abstract 2

The Brachiation model uses only experimentally controllable parameters to predict the rheological behavior of dynamic polymeric materials, greatly enhancing materials design efficiency.

## Introduction

Dynamically associating polymers are a growing field of interest among materials scientists due to their large range of tunability in terms of viscoelasticity and stress relaxation, which are important factors to consider for a number of applications. These dynamic polymer networks are used as self-healing materials for applications where deformation is unavoidable, such as in drug delivery,^1^ biomaterials for cell culture and tissue engineering,^2,3^ rheological modifiers for enhanced oil recovery,^4^ reprocessable plastics,^5^ 3D printing,^6^ and stretchable materials for batteries.^7^ As the underlying physical behavior of these dynamically associating polymer networks dictates their function and suitability for a particular application, it is critical to link molecular-scale design with bulk mechanics and dynamics of the resulting materials. A predictive theoretical model would thus be particularly useful in guiding the design process.

There have been several efforts to provide a direct link between molecular design and material behavior. Rubinstein and Semenov notably proposed a theory for thermoreversible associative behavior in polymers using bond lifetime to describe gelation kinetics that was experimentally tested by Sheridan and Bowman using a model Diels–Alder network involving multiarm polymers linked together.^8−10^ This connection between the mechanical properties (shear modulus) and the kinetic parameters (dissociation rate) was also made by Yount et al. using transient network theory to model linear supramolecular polymers with associative linkers connecting chains.^11,12^ The bulk of the work to provide a predictive model for dynamically associating polymers has centered on multiarm polymers containing associative groups on the ends, such as short multiarm PEG chains with hydrazone bonds or metal–ligand coordination bonds, which allows for the theoretical models, based on some iteration of a Maxwell model (the number of spring and dashpotunits is often dependent on the number of association types), to generally perform well in their predictions.^13−16^ While Maxwell models work well for short multiarm polymer networks with one or two association types, they have limitations when it comes to predicting the behavior of longer linear supramolecular polymers that contain their own chain-associated relaxation modes. Additionally, the time scales for the model are often limited to greater than the time scale of dissociation due to the relaxation process being assigned to a single Maxwell mode.

As an example of a model that accounts for chain relaxation, the slip-link model exhibits satisfactory agreement with rheological properties of linear polymer melts and concentrated solutions of polymers that have fixed points along the chain (called “slip-links”) with effective creation and breakage probabilities.^17^ Nevertheless, there are discrepancies between the theory and experiments, and the model is confined to networks where the dynamic association occurs only at the ends of the chain. The sticky Rouse model, first presented by Baxandall, aims to describe a network of unentangled linear polymers with pendant reversible cross-links.^18,19^ Furthermore, the sticky Rouse model, incorporating relaxation modes inherent to the chain, can be modified to the sticky reptation model to capture the relaxation modes associated with entanglements for entangled, associating polymer networks.^20,21^ Several material systems have experimentally corroborated the sticky Rouse and sticky reptation models, including the dynamics of unentangled and entangled ionomers as well as of entangled silk protein solutions.^22,23^ Although the sticky Rouse model successfully captures the experimental data of dynamically associating polymer networks, this model is phenomenological and incorrectly assumes that the sticker bond lifetime governs the viscoelasticity of the network.^24,25^ Importantly, the sticky Rouse model as well as the Maxwell models used for multiarm polymers with dynamic associations all use a relaxation scheme that scales exponentially with the relaxation time of the association, making an assumption about how the dynamic associations affect chain dynamics.

We have established a molecular-based theory rooted in chain-associated and kinetic parameters that describes the mechanical behavior of linear dynamically associating chains without making any assumptions about how the association kinetics affect the chain dynamics and vice versa.^26^ Modeled on the brachiating motion of gibbons as they travel from tree branch to tree branch in a forest, our model (dubbed the “Brachiation model”) can be leveraged by materials scientists as a predictive tool during the design process for new dynamically associating polymeric materials. The Brachiation model takes as input a range of experimentally controllable characteristics of a physically associating polymeric material, including the polymer molecular weight, the number of “stickers” per chain, the parameters governing the association chemistry, and the polymer concentration. By not assuming how association kinetics affect the chain dynamics, the Brachiation model can predict the frequency-dependent viscoelasticity of the network experienced by each polymer chain across a wide spectrum of frequencies.

**Figure 1:**
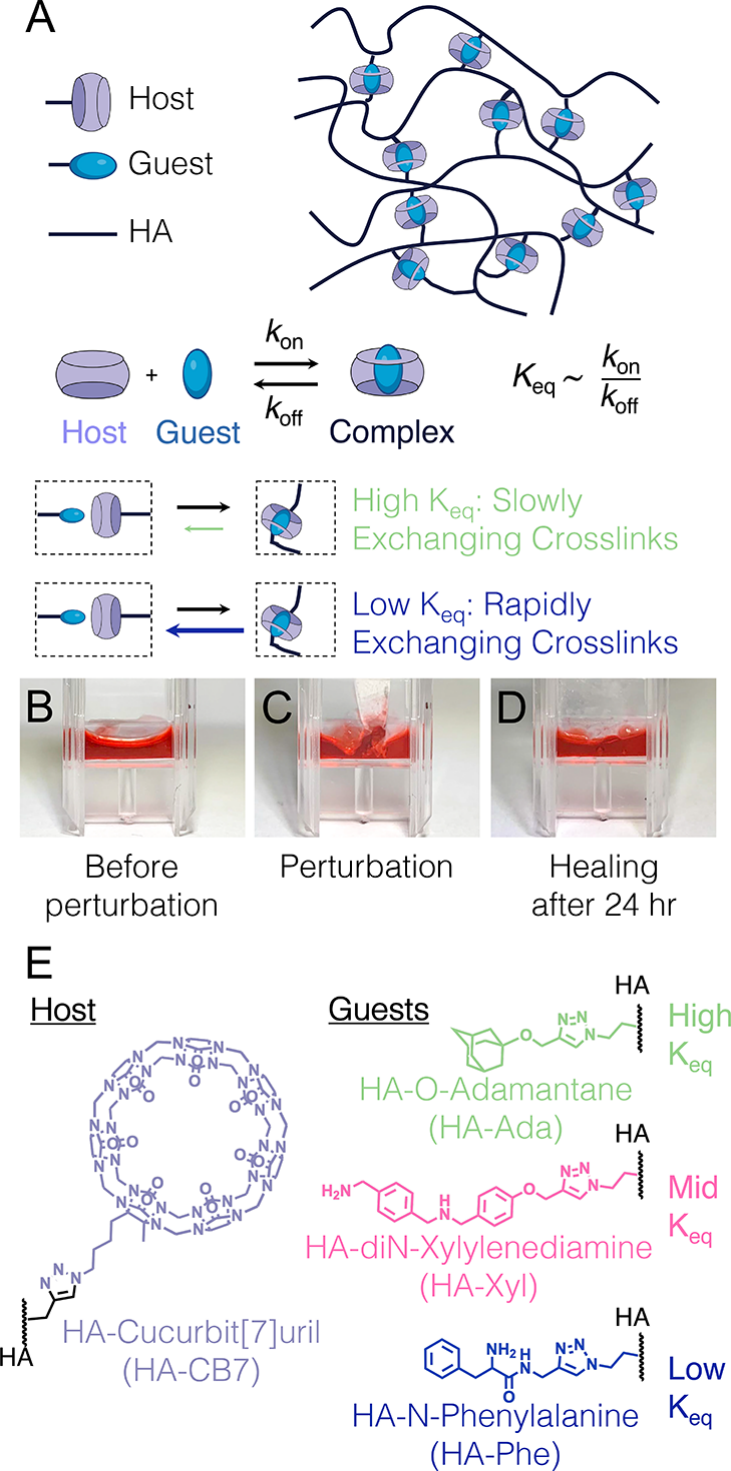
Schematic of the HA host–guest system. Hyaluronic acid (HA, molecular weight 40 kDa) is chemically modified with either guest or CB7 host molecules that transiently form complexes (A). HA host–guest gels were formed (B), then perturbed using a spatula (C), and allowed to heal for 24 h after the perturbation. These dynamically cross-linked gels are self-healing, returning to their original, smooth gel state as before perturbation (D). The equilibrium constants of the CB7–guest complex can be tuned by guest chemistry, with three different guest molecules (HA-Ada, HA-Xyl, and HA-Phe) explored here that bind to the same host molecule (HA-CB7), with HA-Ada having the highest equilibrium constant and HA-Phe having the lowest (E).

In this Article, we use a polymer system composed of hyaluronic acid chains chemically modified with a cucurbit[7]uril (CB7) macrocycle host or corresponding guests to experimentally validate the Brachiation model (Figure 1A). Hyaluronic acid (HA) is a polysaccharide that is ubiquitous in the human body and crucial for many cellular and tissue functions.^27^ Because of its biological significance, many biomedical applications from drug delivery to cell culture scaffolds use HA with clinical success.^28,29^ These applications often use HA polymers modified with dynamically associating groups, a trend that will continue because tissue inherently exhibits the viscoelastic behavior mimicked by physically associating polymer networks. CB7 is a water-soluble macrocyclic host that can bind an array of different guests, with the specific guest chemistry dictating a range of affinity and concomitant dynamics of the host–guest complex. CB7–guest recognition thus has been used to afford dynamic associating interactions in polymer networks.^30^

**Figure 2:**
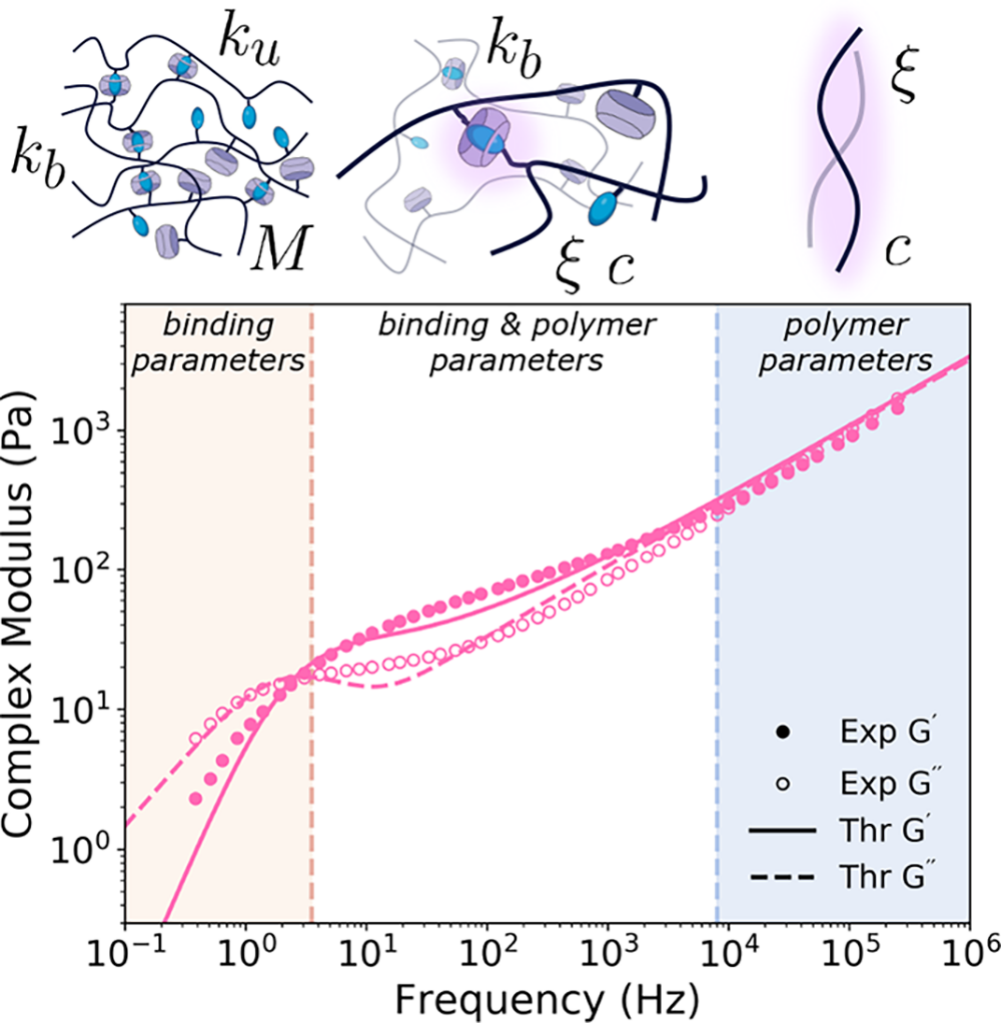
Comparison between rheological prediction by the Brachiation model and experimental data for the 5 wt % HA-CB7 and HA-Xyl gel. The low, middle, and high frequency regimes are highlighted by tan, white, and blue, respectively. The main input parameters of the Brachiation model that dominate rheological prediction in each frequency regime are listed next to graphical representations of the length scale associated with each frequency regime. Additionally, the parameters associated with each regime are labeled as either “binding” (having to do with the associations) or “polymer” (describing the polymer solution). Specifically, *k*_u_ is the unbinding rate constant, *k*_b_ is the binding rate, *M* is the number of association units, ξ is the drag coefficient, and *c* is the polymer concentration. The remaining input parameters to the Brachiation model not listed in the figure are the number of monomers per chain *N*, temperature *T*, and Kuhn length *b*.

Predictions from the Brachiation model cover all frequency regimes of viscoelastic behavior, from the short time scale stress contributions due to local monomer relaxation (blue region in Figure 2) to the intermediate elastic plateau region (white region in Figure 2) to the long time scale full chain relaxation (tan region in Figure 2). To measure a similarly wide frequency range of material behavior experimentally, we use dynamic light scattering microrheology (DLSμR) to measure the rheology across six decades in frequency (10^–6^ to 10 s).^31−37^ This technique leverages the single scattering limit of DLS to track the movement of embedded particles within a polymer network and thereby capture its linear viscoelasticity. DLSμR is a noninvasive microrheology technique that requires a minimum of 12 μL of sample and measures the rheological behavior of materials with stiffnesses in the range of 10^–1^ to 10^4^ Pa. Importantly, our approach does not rely on time–temperature superposition and therefore avoids the assumption that all temperature-dependent processes in these dynamically associating polymer networks have the same temperature dependence.^38^

We have chosen HA as a model polymer backbone and attached the CB7–guest motifs pendant from this backbone affording a range of affinities and kinetic dissociation rates (Figure 1E).^30^ We demonstrate that the inputs of the Brachiation model are the same parameters polymer chemists use to define their synthesized systems, and we further demonstrate the a prior prediction of the viscoelastic behavior of the desired hypothetical material system. Harnessing these capabilities of the Brachiation model can enable the screening of a large parameter space of materials in a time- and cost-effective manner.

## Results

### Theoretical Basis for the Brachiation Model

In our previously published work introducing the Brachiation model, we developed a theoretical framework that captures the viscoelastic behavior of a network of chains along which transient associations can form.^26^ The model considers a single flexible polymer chain of length *N* (number of Kuhn segments) and *M* chemical units (i.e., “stickers”) that can transiently associate with neighboring chains. These associating units are spaced evenly along the chain.

We describe the dynamic motion of the polymer chain using the Langevin equation of motion with a term that includes a viscoelastic memory kernel *K* encompassing the physical forces from the association segment during binding with a neighboring chain in the network.
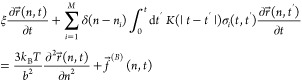
1

The other terms in the governing equation account for the forces from the drag felt by the chain (first term left-hand side), the spring force between the monomers (first term right-hand side), and the Brownian forces felt by the monomers (second term right-hand side). We assume that the bound segment loses all memory of past deformations during association upon unbinding. Thus, the memory kernel *K* links the single-chain dynamics to the surrounding viscoelasticity of the network. The exact analytical form of *K* is determined through a self-consistent calculation of the equation below, enabling the rheological prediction of the complex modulus  for a specific dynamically associating polymer network (Supporting Information).
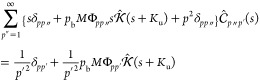
2

The input parameters required to evaluate the Brachiation model are listed in Table 1, of which only the nondimensional unbinding rate constant *k*_u_ (*K*_u_ = τ_R_*k*_u_) appears in eq 2. The other terms in eq 2 include a probability of binding for each association unit (*p*_b_ = *cMk*_b_/(*cMk*_b_ + *k*_u_)), the normal modes *p* used for the normal-mode expansion when solving the Langevin equation, the mode coupling matrix Φ_*pp*′_, which is determined using the number of associating units *M*, and the nondimensional normal-mode correlation function . This implementation of our theory neglects long-range hydrodynamic interactions between segments of the chain (“Brach Rouse” in Table 1). In this current work, we adapt the Brachiation model to capture these long-range hydrodynamic interactions by using a preaveraging approximation (e.g., Zimm model, “Brach Zimm” in Table 1) (Figure S1).^39^

**Table 1:** Brachiation Model Parameters for HA Host–Guest Materials^a^

Host–guest wt %	*c*	*M*	*N*	ξ	*k*_u_	*k*_b_	*T*	Model
CB7–Phe 5%	6.04 × 10^–2^	713	5195	4.05 × 10^–13^	142	1.37 × 10^–4^	25	Brach Zimm
CB7–Xyl 5%	6.04 × 10^–2^	713	5195	5.06 × 10^–11^	1.28	1.73 × 10^–3^	25	Brach Rouse
CB7–Ada 5%	6.04 × 10^–2^	713	5195	5.06 × 10^–10^	0.14	1.92 × 10^–3^	25	Brach Rouse
		
								
CB7–Phe 7.5%	9.07 × 10^–2^	713	5195	5.06 × 10^–13^	142	1.37 × 10^–4^	25	Brach Zimm
CB7–Phe 10%	1.21 × 10^–1^	713	5195	9.22 × 10^–13^	142	1.37 × 10^–4^	25	Brach Zimm
CB7–Xyl 3%	3.63 × 10^–2^	713	5195	4.25 × 10^–12^	1.28	1.73 × 10^–3^	25	Brach Zimm
CB7–Xyl 7.5%	9.07 × 10^–2^	713	5195	7.60 × 10^–11^	1.28	1.73 × 10^–3^	25	Brach Rouse
CB7–Ada 0.25%	3.02 × 10^–3^	713	5195	4.05 × 10^–12^	0.14	1.92 × 10^–3^	25	Brach Zimm
CB7–Ada 3%	3.63 × 10^–2^	713	5195	5.06 × 10^–11^	0.14	1.92 × 10^–3^	25	Brach Rouse

a The Brachiation model parameters of polymer concentration *c* (M^–1^), number of stickers per chain *M*, number of monomers per chain *N*, drag coefficient ξ (kg/s for Brach Rouse, Pa·s for Brach Zimm), unbinding rate *k*_u_ (s^–1^), binding rate *k*_b_ (M^–1^ s^–1^), and temperature *T* (°C) were fitted to the 5 wt % rheological data of each host–guest pair (top three rows, above line). The theoretical predictions for the remaining concentrations for each host–guest pair were determined by altering the drag coefficient ξ and the concentration parameter *c* in proportion to the experimental difference in polymer concentration (below the double lines). The model describes whether hydrodynamic effects were included to make the prediction, where Brach Zimm was chosen for when the material was predicted to be in the sol phase.

The Brachiation model is able to predict rheological behavior over the low, intermediate, and high frequency regimes (solid line in Figure 2) due to its incorporation of all modes of relaxation in the network and its self-consistent memory kernel structure. The rheological behavior in each frequency regime is dominated by a different set of molecular parameters from the model. For the high frequency regime, the polymer-centric parameters of concentration *c* and drag ξ dominate this short time scale and length scale behavior. In the low frequency regime, the binding-related parameters of unbinding rate *k*_u_, binding rate *k*_b_, and number of associating units *M* dominate the behavior. The kinetics of the binding govern the network connectivity so the terminal relaxation of the entire network is expected to depend heavily on the binding parameters.

### Dependence of HA Host–Guest Material Rheology on Concentration and Dissociation Kinetics

**Figure 3:**
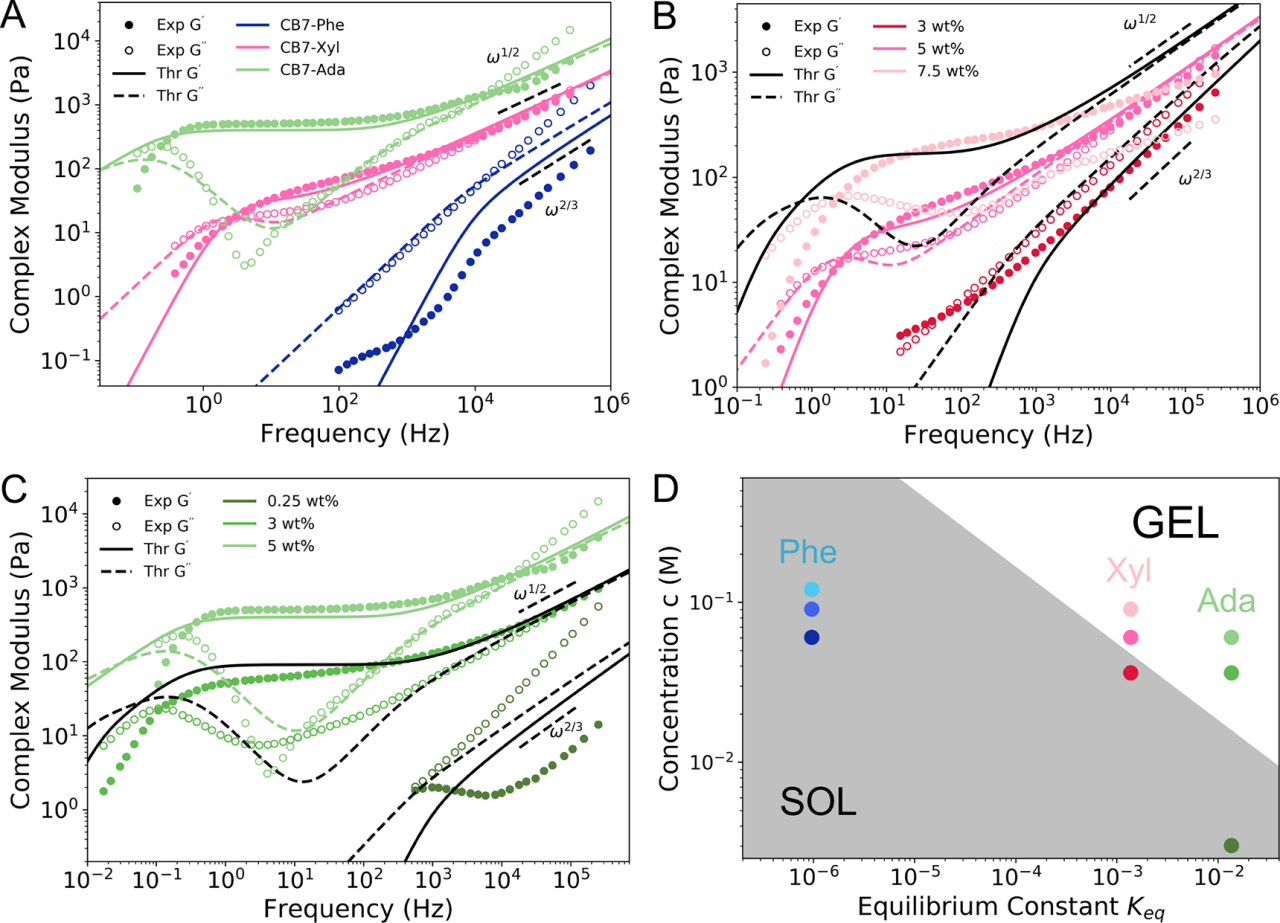
Rheological measurements and Brachiation model predictions of HA host–guest polymers. The measured rheological values of all three CB7–guest pairs at a polymer concentration of 5 wt % are shown along with the theoretical rheological values (A). The measured rheology of HA-CB7 and either HA-Xyl (B) or HA-Ada (C) gels at varying polymer concentrations is shown along with the theoretical rheological predictions. Black lines are predictions, while colored lines are fits to the experimental data. The theoretical sol–gel phase diagram (gray/white) allows the prediction of the phase behavior of each HA CB7–guest pair at varying polymer concentrations, which can be directly compared to the experimental observations (colored dots) (D).

HA is a linear polymer containing chemically modifiable carboxylic acid groups along its backbone. These side groups are chemically modified with guest and host molecules to form the HA host–guest material system. The following two separate modifications are made: (1) HA modified with the host molecule, cucurbit[7]uril (CB7), and (2) HA modified with the guest molecule, which is one of three options: adamantane (Ada), xylylenediamine (Xyl), and phenylalanine (Phe) (Figure 1A). The dynamic CB7–guest associations give self-healing properties to the HA-based material, as exhibited by the return of a 5 wt % HA-CB7 and HA-Ada mixture to a smooth gel 24 h after perturbation with a spatula (Figure 1B–D). The three CB7–guest pairs studied have different equilibrium constants (*K*_eq_), similar association rates (*k*_on_), and different dissociation rates (*k*_off_), with the CB7–Ada pair offering the lowest dissociation rate and the CB7–Phe pair having the highest (Figure 1E).^30^ A higher *K*_eq_ thus corresponds to a slower cross-link exchange, with more long-lived cross-links creating a more elastic network and slowing stress relaxation (Figure 1A). Rheological measurements of the HA polymer networks with each of the CB7–guest pairs confirm the physical implications of the different kinetic dissociation rates. For the same polymer concentration (5 wt %) and degree of modification (20%), the highest *K*_eq_ pair (CB7–Ada) exhibits the greatest complex modulus value, and the lowest *K*_eq_ pair (CB7–Phe) exhibits the lowest complex modulus values across all frequencies (Figures 3A and S4–S7).

Another tunable parameter of these HA host–guest materials is the polymer concentration. The rheology of the CB7–Xyl (intermediate *K*_eq_ value) and CB7–Ada (highest *K*_eq_ value) systems across a range of concentrations demonstrates that, as the concentration increases, the viscoelasticity of the material increases, while lower polymer concentrations exhibit more viscous behavior and less elastic contribution (Figure 3B,C). Over the polymer concentration range of 3–5 wt %, the HA CB7–Xyl pair undergoes a change in the high frequency scaling regime (Figure 3B). At a concentration of 5 wt % in the CB7–Xyl pair (bright pink), the scaling in the high frequency follows *G*′∼ ω^1/2^. Once the concentration decreases to 3 wt % (dark red), the scaling in the high frequency transitions to *G*′∼ ω^2/3^. A scaling of ω^1/2^ in the high frequency region corresponds to the Rouse model scaling, which assumes that at small length scales the monomer–monomer interactions dominate the physical dynamics of the polymer chains.^39^ However, a scaling of ω^2/3^ in the high frequency region corresponds to the Zimm model scaling, which assumes that at small length scales the hydrodynamic interactions between the solvent and each monomer dominate the physical behavior. These small length scales where hydrodynamic interactions dominate the physical behavior are defined by the correlation length, which can be estimated for the 3 wt % polymer concentration to be 22 nm. This length scale can be related to a frequency by finding the inverse of the relaxation time corresponding to the *p*th mode of the *N*/*p* number of monomers within a correlation length. The frequency corresponding to the correlation length is found to be 2.5 × 10^4^ Hz, which agrees with the frequency above which we observe experimentally the 2/3 scaling and indicates the frequency above which the Zimm model should be used to accurately account for the hydrodynamic interactions. As the polymer concentration decreases, shifting from the semidilute to dilute regime, we expect to see a more dominant role played by hydrodynamic interactions on polymer dynamics due to a lower density of chains and a greater necessity to incorporate long-range hydrodynamics (i.e., Zimm).^39^

Furthermore, if we look at Figure 3A, as the dissociation rate increases while holding the polymer concentration constant, we also see a change in the high frequency scaling. We observe that in systems where the dissociation rate is high, such as in the CB7–Phe pair, the short time scale behavior follows *G*′∼ ω^2/3^. In the lower dissociation rate pairs, such as CB7–Ada, we see that the rheological behavior in the short time scale follows *G*′∼ ω^1/2^. These observations indicate that those short-lived associations (e.g., CB7–Phe) lead to local dynamics that are more dominated by hydrodynamic interactions due to the frequency scaling. We argue that, in the case of the lower dissociation rate pairs, these longer-lived associations exhibit considerably more dynamic heterogeneity with local chain dynamics forming many different connections with other chains, both locally and distantly. This heterogeneity, in turn, is hypothesized to screen hydrodynamic interactions and contributes to increased viscoelasticity, a phenomenon reminiscent of how the surface heterogeneity of colloids can increase the viscoelasticity of colloidal suspensions.^40^

### Brachiation Accurately Predicts the Rheological Behavior of HA Host–Guest Materials

The Brachiation model takes as input parameters pertaining to the molecular-level description of a dynamically associating polymer network (listed in the Table 1 caption). Beginning with the host–guest pair of intermediate *K*_eq_ value, CB7–Xyl, the parameters of the Brachiation model (*c*, *N*, *M*, *k*_u_, *k*_b_, and ξ) were fit to the experimentally determined rheological data of the 5 wt % solution using a simulated annealing algorithm. We find that the parameters scale well with the experimental values. For example, the ratio of the fitted number of monomers *N* to the fitted number of stickers *M* is 5195 to 713. On the basis of our NMR integration, the degree of modification for these polymers should put that ratio experimentally at 5:1, which is similar to our fitted value of 7:1 (Figures S4–S7). Using these fitted parameters, we compared the Brachiation model prediction to the experimentally measured rheological output. Across all three frequency regimes, the Brachiation model prediction displays good agreement with the experimentally measured rheological spectrum of the HA gel prepared from CB7–Xyl cross-linking at 5 wt % (Figures 2 and 3B, pink lines). In the high frequency region, the scaling of *G*′∼ ω^1/2^ is captured. In the intermediate region, the shape of *G*′ and *G*^″^ fits the data well. In the low frequency region, the expected terminal scalings of *G*′∼ ω^2^ and *G*^″^∼ ω are recovered.^39^

Next, by altering the concentration parameter *c* by the proportional values of 7.5/5 and 3/5 and the drag coefficient ξ, we obtain the rheological predictions from the Brachiation model for HA-CB7 and HA-Xyl networks at 7.5 and 3 wt %, respectively (Figure 3B, black lines). In the case of the 7.5 wt % concentration, we see reasonable agreement between the theoretical prediction and the experimental measurement. For the 3 wt % concentration, as mentioned earlier, the scaling in the high frequency changes to ω^2/3^, so this fit necessitated a switch to the Zimm treatment of the monomer level interactions to be able to fully describe the hydrodynamic effects observed experimentally. Using the Zimm treatment, we see very good agreement between the experimental measurement and theoretical prediction in the scaling and magnitude of the complex modulus for the 3 wt % solution of HA-CB7 and HA-Xyl. To demonstrate reproducibility of this approach, we also show similar results for theoretical fit (Figure 3C, green lines) and theoretical predictions for the CB7–Ada (Figure 3C, black lines) and CB7–Phe host–guest systems (Figure S10). As with the CB7–Xyl analysis, we find excellent agreement between our theoretical predictions and the experimental measurements across all three frequency regimes (Figure 3C).

These two versions of the Brachiation model (Rouse and Zimm) fully capture the sol to gel phase transition of the HA network prepared from CB7–guest cross-linking. We define the gel phase by the existence of a minimum in the plot of the tangent delta [tan(δ) = *G*′/*G*^″^] (Figures S11–S13). The hallmark features of the phase transition occur across each of the different frequency regimes, from the scaling change in the high frequency to the loss of the elastic plateau in the intermediate region in the sol phase. Thus, measuring across this broad spectrum of frequencies experimentally necessitates a technique like DLSμR that can capture the low to high frequency behaviors without the use of time–temperature superposition. While time–temperature superposition is invaluable for the analysis of polymer solutions, the inherent assumption that a change in temperature leads to a commensurate change in all processes in the system, such as chain relaxation and physical association rate, is invalid for a polymer network with dynamic associations.^10,25^ For example, for the HA CB7–guest system, the kinetics of association are expected to follow an Arrhenius dependence on temperature, which is distinct from the temperature dependence for the full chain relaxation dynamics.^41^

To investigate the prediction of the Brachiation model across a range of kinetic parameters, we used the same polymer and concentration parameters (*c*, *N*, *M*) across all host–guest pairs and altered the kinetic parameters (*k*_u_, *k*_b_). As expected, the fitted equilibrium constant (*K*_eq_ = *k*_b_/*k*_u_) for CB7–Ada, which has the highest experimentally determined *K*_eq_, is also the highest out of the three host–guest pairs and is followed by that found for CB7–Xyl and then that found for CB7–Phe, the pair with the lowest experimentally determined *K*_eq_ (Figure 1 and Table 1).^30^ Additionally, we see that in the high frequency region of the 5 wt % HA-CB7 and HA-Ada, *G*′ scales with ω^1/2^, validating our choice of using the Rouse treatment for predicting the rheological behavior of this system. For the CB7–Phe pair at 5 wt %, the high frequency behavior deviates from the Rouse scaling and tends to a higher scaling, which we can account for by altering the monomer length scale treatment to be Zimm-like. Altogether, we show that the Brachiation model can correlate kinetic parameters describing the associating units to the rheological spectrum for a family of polymeric materials across a range of polymer concentrations, a range of association kinetics, and both gel and sol phases.

## Discussion

The results shown here support the idea that the Brachiation model can be used by polymer chemists as part of the design process when creating new materials for a specific application. For example, control of complex modulus is a common goal when designing hydrogels for biomaterials applications, as cells are well-known to respond to the viscoelasticity of their surrounding environment.

The Brachiation model serves as a predictive framework to tailor the viscoelasticity of an HA host–guest polymer network, guiding the selection of specific host–guest pairs and informing the optimal polymer concentration for the desired viscoelasticity. This capability of the Brachiation model hinges on it being a molecular-based model. The model does not coarse-grain the polymer network but rather takes in the molecular-level descriptors (number of monomers *N*, number of associating units *M*, and polymer concentration *c*) and binding parameters (unbinding rate constant *k*_u_ and binding rate constant *k*_b_) of a single polymer and self-consistently evaluates the dynamics of a network of such polymers. Additionally, the model uses a self-consistent framework that does not constrain network relaxation to depend exponentially on the dynamic association lifetime.^13−16,18,22^

Despite the general agreement between the theoretical predictions and experimental measurements, Figure 3 still exhibits some discrepancies between experiment and theory. One of those discrepancies is the lack of alignment in the intermediate and high frequency regimes of the complex modulus. In part, the lack of alignment stems from limitations in DLSμR, which leverages the correlation function of scattering patterns from DLS to evaluate the complex modulus. In the high frequency regime, which corresponds to the short time scale and length scale behavior, the measurement is limited by the short-time behavior of the correlation function. There is a limit to the degree of movement by the probe particles within the sample that can be reflected by the correlation function. Take, for example, a material with significant elastic contribution to the complex modulus. A particle embedded in such a network would not move very far within short times due to the elastic nature of the network resisting the particle’s Brownian motion away from its starting position. If the correlation function registers minimal change at short time scales due to the limited capability of the instrument to detect the small physical movements of the particle, we expect the scaling in the high frequency of the complex modulus to be lower than even Rouse scaling (i.e., for G*∼ ω^α^, α < 1/2). This is observed in the high frequency scaling for the CB7–Xyl system at 7.5 wt % (Figure 3B). Alternatively, a particle favorably interacting with the network (i.e., electrostatic attraction) would also exhibit a smaller range of movement, but we have shown that the particles used here are not interacting with the polymers (Supporting Information).^32^ Last, the numerical Fourier transform used to obtain the complex modulus in DLSμR is subject to error in the short time limit of the correlation function, potentially explaining the much higher slope of the experimental data in the high frequency region for materials in the sol phase (Figure 3C, 0.25 wt % curves).

In the intermediate frequency regime, we also see a discrepancy between the predicted and experimental values in the dip of the *G*^″^ for gel phase systems. DLSμR leverages the scattering correlation function to obtain a mean-squared displacement of embedded probe particles, which is then translated into the complex modulus using the generalized Stokes–Einstein equation. Because the value of *G*^″^ is orders of magnitude lower than *G*′ in these intermediate frequencies of a gel, any noise in the scattering correlation data will become more magnified in the value of *G*^″^ than in *G*′. Thus, we generally see better agreement between experiment and theory for *G*′ than *G*^″^ at these intermediate frequencies. Additionally, due to the fast gelation time of some of the host–guest pairs, there could be heterogeneity within these materials during rheological characterization using DLSμR. This heterogeneity was detected by measuring the scattering intensity as a function of position within the material and accounted for using a previously published adjustment factor.^37^ We have also performed macrorheology on the 5 wt % CB7–Xyl material at a single temperature, which has a limited range of frequency (Figure S8). Within this frequency range, the two rheology techniques agree in magnitude and shape of the complex modulus. The shift in the crossover frequency could be due to a combination of temperature variances and heterogeneity in the sample (Figure S9).

The Brachiation model imposes assumptions about the dynamic polymer network, and some assumptions could result in the observed discrepancies. First, we assume that there is no entanglement in the network, which is corroborated by estimations of the critical entanglement concentration for 40 kDa HA being above our maximum polymer concentration of 10 wt % (Supporting Information).^42^ The Brachiation model also assumes that associating units are evenly distributed along the chain, but this geometry was not verified experimentally and could be untrue for some of our polymers. We believe this would lead to a lower modulus if the stickers were highly skewed toward one end of the chain. That being said, the number of stickers per chain was experimentally validated and probably evenly distributed (Figures S4–S7). While the Brachiation model does not impose an explicit exponentially decaying relationship between the associating unit and network relaxation, it does assume that the probability of binding decays exponentially with time bound and that once an associating group unbinds it loses all memory of the stress born by the association. Both of these assumptions can contribute to the discrepancy seen as the assumption that the binding process follows a Poisson process, although a good approximation, is only a guess at how the binding process proceeds.^26^ Moreover, the idea that an associating unit can unbind but then rebind again immediately to the same partner and thus still contribute to the network viscoelasticity has been explored by van Ruymbeke and co-workers and is a scenario that further development of the Brachiation model should explore.^43^

The Brachiation model employs molecular-level parameters, but not all of the model input parameters are as straightforward to obtain when describing a dynamic polymer network. For example, the drag coefficient ξ is a parameter that encompasses an inherent physical property of the polymer network but can be challenging to experimentally or theoretically determine. For example, the effective drag coefficient ξ of a polymer network at a higher polymer concentration may not be equivalent to the drag coefficient of a lower polymer concentration solution. Thus, as far as adjusting parameters to obtain accurate rheological predictions of hypothetical polymer materials, it is important to keep in mind that the drag coefficient affects the model’s output. While the theoretical predictions in Figure 3 include altered drag coefficients for each host–guest pair and polymer concentration, we find that not altering the drag coefficient parameter results in a slight frequency shift in the rheological spectra but does not predict a different phase of the material (i.e., sol vs gel) (Figures S14–S16).

To examine the experimental relevance of our theory parameters from the Brachiation model to the polymer system, we examine the parameters related to the polymer. As mentioned above, the fitted parameters of the number of monomers *N* and associating units *M* imply a degree of modification of the polymer chain similar to that found experimentally (Figures S4–S7). A higher degree of modification (i.e., increasing *M* while keeping *N* constant) should lead to more elasticity in the network due to more sites of binding along each chain. The concentration parameter *c* is intentionally kept proportional to the experimental polymer concentration for each polymer material (Table 1). Next, we compare the theoretical values for the equilibrium constant *K*_eq_ to the experimentally determined values. The theoretically determined values for the equilibrium constant for the different host–guest pairs are 1.35 × 10^–2^, 1.36 × 10^–3^, and 9.63 × 10^–7^ for CB7–Ada, CB7–Xyl, and CB7–Phe, respectively. The experimentally determined equilibrium constants for each host–guest pair when free in solution were previously reported to be 2.6 × 10^10^, 1.3 × 10^9^, and 1.5 × 10^7^ for CB7–Ada, CB7–Xyl, and CB7–Phe, respectively.^30^ We observe that the theoretically determined values for the probability of binding scale proportionally with the measured equilibrium constants for each host–guest pair, although we do see a slight deviation between theory and experiment for the *K*_eq_ of the CB7–Phe pair. We also notice that the absolute values of *K*_eq_ differ greatly between theory and experiment. This discrepancy could be a result of how we have defined the probability of binding in the theory. That *K*_eq_ from the Brachiation model scales proportionally with the experimentally determined *K*_eq_, while maintaining the values of the chain descriptors (*c*, *M*, *N*), is an advantage over the capabilities of the sticky Rouse model. While the sticky Rouse model requires fewer parameters and exhibits a remarkably good fit to the data, the parameters that are found to best fit the experimental data do not scale accordingly with the respective experimental parameter (Figures S17 and S18). For example, when we fit the CB7–Ada pair rheological data at 3 and 5 wt % to the sticky Rouse model, we see the association relaxation time τ_S_ jump from 4.72 × 10^–10^ to 3.20 × 10^–4^ even though the associating unit’s affinity is still the same (Table S3).

We can use the association parameters and polymer concentration to predict the phase of a dynamic polymer network using our Brachiation model (Figure 3D). Keeping all parameters constant besides the ones varied along the two axes (concentration *c* and equilibrium constant *K*_eq_), we create a sol–gel phase diagram where the dividing line scales as . We show this sol–gel phase diagram as a direct experimental demonstration of the previously published phase diagram^26^ but acknowledge that it does not encompass coincident thermodynamic phase behavior quantities, such as the spinodal transition line, that are equally important to the internal structure of dynamic polymer networks.^44−46^ Future work includes investigating the convergence of the thermodynamic and sol–gel phase diagrams for these networks. In the sol–gel phase diagram determined with the parameters for the HA host–guest polymers, we see that the theoretically predicted points fall into the correct phases in Figure 3D. For the lowest affinity pair, HA with CB7–Phe cross-links, these polymer networks remain in the sol phase. For the medium affinity pair, HA with CB7–Xyl cross-links, polymer concentrations above 5 wt % result in the gel phase, which agrees with our experimental rheological observations. Last, for the highest affinity pair, HA with CB7–Ada cross-links, polymer concentrations above 0.25 wt % result in the gel phase, agreeing with the experimental results. For the same polymer concentration, increasing the association probability from low equilibrium constant to high equilibrium constant transitions the polymer network from the sol to the gel phase (left to right along the 5 wt % concentration). The ability to accurately predict the phase of a dynamic polymer network based on the molecular-level parameters that describe the network is extremely useful for quickly screening many iterations of a dynamic polymer system before synthesizing the one that will best meet the needs of a specific application.

The rheological behavior of a dynamic polymer network depends not only on the polymer concentration and the association dynamics but also on the chain length and the number of associating units per chain. Whether the influence of the chain length and number of associating units on rheological behavior is consistent between the Brachiation model and experiments remains to be verified. As with many polymeric systems, the HA chain length typically spans a broad range and thus is not well suited for experimental verification of chain length effects (Table S1). Thus, to test whether the chain length and number of associating units affect rheology in the same manner between theoretical and experimental results, our dynamic polymer system must exhibit a significant degree of control over chain length and, consequently, number of associating units per chain.

Geometries of dynamic polymer networks that can be well-predicted by the Brachiation model are currently limited to linear chains with pendant associations. This geometry is very common in dynamic polymer networks as many synthetic and naturally derived polymers are linear and have chemical groups that can be modified along the chain.^47−50^ It is worth noting that the sticky Rouse model has been shown to agree with the experimental data of dynamically associating polymers of a variety of geometries.^51,52^ This result, however, is possible due to the use of an effective friction in the model that represents the slower relaxation modes associated with the stickers on the chains but is not defined by any molecular-level kinetic parameters.^9,53,54^ To properly describe the geometries beyond a linear polymer chain, the Brachiation model requires further modification to account for additional architectural complexity of the polymer constituents. A downside to sticky Rouse and any other Maxwell-type model is its inability to be adapted to predict the nonlinear rheological behavior of these dynamically associating networks. The Brachiation model, however, has the framework to be adapted to predict nonlinear behavior due to its self-consistent nature, and we see this development as a future direction for the model.

**Figure 4:**
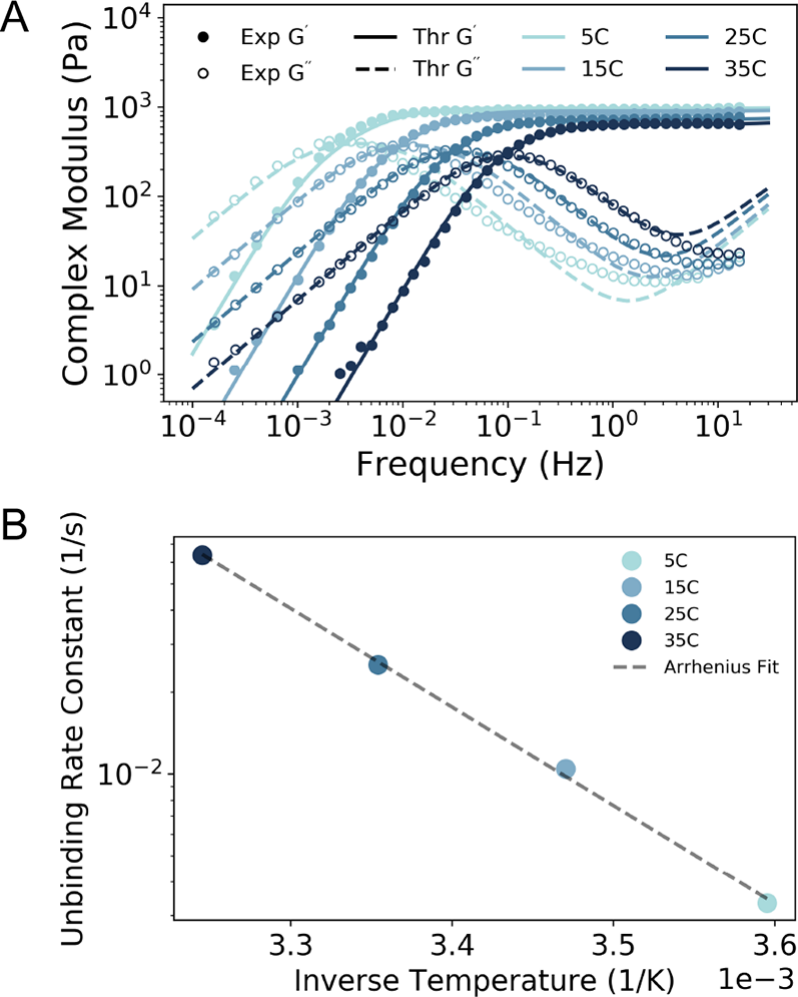
Rheological measurements and theoretical predictions of histidine–nickel poly(*N*,*N*-dimethylacrylamide) (PDMA) polymers. The measured rheological spectra (data reproduced here from Tang et al.^55^) and theoretical predictions from the Brachiation model for PDMA dynamically associating via histidine–nickel bonds are shown for four different temperatures (A). The fitted unbinding rate constant is plotted as a function of inverse temperature, and an Arrhenius fit line is shown as a comparison to the points (B).

For polymer systems that are linear with associations along the chain, the Brachiation model can predict the behavior of polymers with a wide range of associations. Many different types of transient associations have been explored by polymer chemists, including dynamic covalent bonds and metal–ion coordination bonds.^55,56^ The Brachiation model can be used to describe the rheological behavior of these networks. As an example, data taken from a polymer system made up of poly(*N*,*N*-dimethylacrylamide) (PDMA) polymers associating via histidine–nickel bonds were fit using the Brachiation model (Figure 4A). We find that the fitted parameters *N* and *M* scale similarly to the experimentally determined fraction of stickers with *M*/*N* ≈ 2% and the actual system at 5.3% (Table S4). This particular polymer system is highly temperature-sensitive and will exhibit not only shifts in the viscoelasticity but also terminal relaxation time as a function of temperature.^55^ Plotting the fitted unbinding rate constant against inverse temperature, we see that the fitted unbinding rate *k*_u_ follows the Arrhenius equation (Figure 4B). These results show that the Brachiation model is able to separate the contributions from the bond association kinetics and the chain relaxation to the overall network elasticity. Furthermore, the fit to the data when separating these contributions underscores the benefit of the Brachiation model, which does not assume time–temperature superposition, for dynamic polymer networks consisting of temperature-dependent processes that each scale uniquely with temperature.

## Conclusion

Dynamic polymer networks are ubiquitous across many different fields of science, but the design process for these networks is still complex given a broad parameter space that includes a range of associations, polymer types, and phase behavior. The Brachiation model has been shown here to capture the essential rheological behavior of an associating polymer system as described by its molecular-level parameters. Such a model will prove useful for polymer chemists who aim to explore and screen a large range of polymer materials quickly for their desired physical behavior. The rheological behavior of dynamic polymer networks, including the ability to form gel networks, is often a key criterion that dictates their function and utility. Because the Brachiation model relies on molecular-level, experimentally measurable parameters, experimentalists are able to directly translate the model’s parameters to an experimental system that would give the predicted rheological behavior. This theoretical model does not rely on assumptions about the temperature dependence of processes within the polymer network nor does it ignore the frequency-dependent viscoelastic response of the network, enabling its rheological predictions to be consistent with experimental values. Further studies to test the influence of chain length and number of associating units on predicted rheological behavior and modifications to fit other geometries are still required to better generalize this model. As currently presented, the Brachiation model offers a highly useful tool in efficiently discovering and evaluating new dynamically associating polymeric materials.

## Supporting Information Available

Experimental procedures and characterization data for all new compounds, materials and methods, Figures S1–S8, and Tables S1–S4 (PDF)
