# Supplementary material for: Rheological Characterization and Theoretical Modeling Establish Molecular Design Rules for Tailored Dynamically Associating Polymers
Source: ACS Cent Sci. 2022 Sep 12;8(9):1318–27. doi: 10.1021/acscentsci.2c00432 (PMC9523779; doi:10.1021/acscentsci.2c00432)
Supplement: Supplementary file 1 — oc2c00432_si_001.pdf [file oc2c00432_si_001.pdf]

Supporting Information for

Rheological Characterization and Theoretical Modeling Establish Molecular Design Rules for Tailored Dynamically Associating Polymers

Pamela C. Cai, Bo Su, Lei Zou, Matthew J. Webber, Sarah C. Heilshorn, Andrew J. Spakowitz

Corresponding Author: Andrew J. Spakowitz.
E-mail: ajspakow@stanford.edu

This PDF file includes:

Supporting text
Figs. S1 to S18
Tables S1 to S4
SI References

Supporting Information Text

Conversion of the memory kernel to the complex modulus. In the Brachiation model, we use a self-consistent calculation to derive the memory kernel $\hat{\mathcal{K}}$. From here, we can use Equation 2 from the main text (reproduced below)

$$\sum_{p''=1}^{\infty} \left\{ s\delta_{pp''} + p_b M \Phi_{pp''} s\hat{\mathcal{K}}(s+K_u) + p^2 \delta_{pp''} \right\} \hat{\mathcal{C}}_{p''p'}(s) = \frac{1}{p'^2}\delta_{pp'} + \frac{1}{p'^2} p_b M \Phi_{pp'} \hat{\mathcal{K}}(s+K_u). \quad [1]$$

to derive the correlation function $\hat{\mathcal{C}}_{p''p'}(s)$. We then use the correlation function to derive the complex modulus in frequency space using the following equation.

$$\frac{N\tilde{G}(\omega)}{ck_BT} = i\tau_R\omega \sum_{p=1}^{\infty} p^2 \hat{\mathcal{C}}_{pp}(i\tau_R\omega) \quad [2]$$

The last step is to dimensionalize the left hand side so that the complex modulus has units of Pascals.

Brachiation Model Accommodates Many Types of Short Lengthscale Behavior in Dynamically Associating Networks. In our previously published work introducing the Brachiation model, the short lengthscale behavior followed the Rouse treatment, neglecting long-range hydrodynamic interactions between segments of the polymer (1). However, our goal is to predict the rheological behavior of polymer networks under a range of concentrations and kinetic parameters, including dilute conditions where chain dynamics are strongly impacted by hydrodynamic interactions. Thus, the need to account for the change in the dominant physical interactions at short lengthscales necessitates a modification to the original Brachiation model. Specifically, we exchange the original Rouse treatment

$$\mathbf{H}_{nm} = \frac{\mathbf{I}}{\xi}\delta_{nm} \quad [3]$$

for a pre-averaging approximation (*e.g.* the Zimm model)

$$\langle \mathbf{H}_{nm} \rangle_{eq} = \frac{\mathbf{I}}{(6\pi^3|n-m|)^{1/2}\eta_s b} \quad [4]$$

to capture the long-range hydrodynamic interactions (2). These two treatments result in two different scalings in the high frequency regime (region above the frequency of 10^3 Hz in Figure S1), with each treatment exhibiting the expected scaling values (Zimm scales as $G' \sim \omega^{2/3}$, and Rouse scales as $G' \sim \omega^{1/2}$). Since these two treatments only pertain to the dynamics of the small lengthscale behavior, the predictions from the Brachiation Model for all other frequency regimes remain the same as demonstrated by the behavior in the frequencies below 10^3 Hz in Figure S1.

To toggle between these two versions of the Brachiation Model as shown in Figure S1, the unbinding rate of the Rouse version is translated by a factor of τ_r/τ_z, where

$$\tau_r = \frac{\xi N^2 b^2}{3\pi^2 k_B T} \quad [5]$$

$$\tau_z = \frac{\eta_s (N^{1/2} b)^3}{\sqrt{3\pi} k_B T}, \quad [6]$$

to obtain the unbinding rate of the Zimm version and the new scaling of $\omega^{2/3}$ in the high frequency regime with all else the same.

Estimating Entanglement Concentration for 40kDa Hyaluronic Acid. We use the following relationship for the entanglement volume fraction ϕ_e (3):

$$\phi_e = \left(\frac{N_e}{N}\right)^{3\nu-1} \quad [7]$$

where N_e is the number of monomers between entanglements, N is the total number of monomers per chain, and ν is the solvent quality parameter. We use the scaling in the above equation to determine the entanglement volume fraction for our specific polymer and molecular weight with the following relationship:

$$\phi_{e,2} = \left(\frac{N_1}{N_2}\right)^{3(0.588)-1} \phi_{e,1} \quad [8]$$

where N_1 and N_2 are 2 different molecular weights of the same polymer type and $\phi_{e,1}$ and $\phi_{e,2}$ are the respective entanglement volume fractions. We use the good solvent value of 0.588 for ν and a value of 10 g/L for a hyaluronic acid chain of molecular weight 1.43×10^6 g/mol to estimate an entanglement concentration for 40kDa hyaluronic acid in 1X PBS of 154 mg/mL, which is greater than our highest polymer concentration of 100 mg/mL (4).

Materials and Methods

HA-alkyne synthesis. Hyaluronic acid (Lifecore Biomedical, 40kDa M_w) is reacted in DMSO for 24 hours with 1.5eq 1-Ethyl-3-(3-dimethylaminopropyl)carbodiimide (EDC) (Thermo Fisher), 1.5eq N-hydroxysuccinimide (NHS) (Thermo Fisher), 3eq 4-methylmorpholine (Sigma Aldrich), and 1.5eq propargylamine at room temperature. The product is dialyzed (3kDa MWCO) for 3 days before lyophilization. NMR analysis of the final product was performed for verification (Figure S2).

HA-azide synthesis. Hyaluronic acid (Lifecore Biomedical, 40kDa M_w) is reacted in DMSO for 24 hours with 1.5eq 1-Ethyl-3-(3-dimethylaminopropyl)carbodiimide (EDC) (Thermo Fisher), 1.5eq N-hydroxysuccinimide (NHS) (Thermo Fisher), 3eq 4-methylmorpholine (Sigma Aldrich), and 1.5eq azido-propylamine (Click Chemistry Tools) at room temperature. The product is dialyzed (3kDa MWCO) for 3 days before lyophilization. NMR analysis of the final product was performed for verification (Figure S3).

HA-cucurbit[7]uril synthesis. CB7[7]-N_3 was synthesized according to previously published methods (5). The pure CB[7]-N_3 product was reacted with HA-alkyne and clicked on via copper-catalyzed azide-alkyne click chemistry following a previously published procedure (6). After click chemistry, the product was dialyzed (3kDa MWCO) for 3 days before lyophilization. NMR analysis of the final product was performed for verification (Figure S4).

HA-Guest synthesis. Alkyne-linked adamantane, p-xylylenediamine, and phenylalanine small molecule guests were synthesized according to previously published methods (6). HA-azide was modified with each of these guests via copper-catalyzed azide-alkyne click chemistry following previously published procedures. After click chemistry, the product was dialyzed (3kDa MWCO) for 3 days before lyophilization. NMR analysis of the final products were performed for verification (Figure S5-S7).

PEGylation of polystyrene probe particles. 500-nm polystyrene microspheres (Polysciences) were PEGylated using a previously published procedure (7, 8). Successful PEGylation was verified using a dynamic light scattering instrument to detect change in particle diameter from the addition of the PEG chains coating the particles (Table S2). The 500nm diameter was verified to be greater than the estimated mesh size of the network (9nm). Additionally, minimization of particle-sample interaction using PEGylation was previously tested and found to be true for this particular PEG molecule and HA host–guest material (7).

Dynamic Light Scattering Microrheology (DLSμR) of HA Host–Guest Materials. To make each HA host–guest mixture, the lyophilized HA material is dissolved in 1X PBS (pH 7.4) at the desired concentration and rotated at room temperature (25°) overnight. Next, 500-nm polystyrene particles PEGylated with 750Da methyl-terminated PEG (Sigma #07964) are mixed into each component, HA-CB7 or HA-Guest, at 0.1% (v/v). The two components at the desired polymer concentration are mixed together to a final volume of 40μL by pipetting up and down, then transferred to a cuvette and capped (Malvern #ZEN0040). After an hour to allow the mixture to reach equilibrium, the cuvette containing the polymer mixture is probed at 25°C using a dynamic light scattering instrument (Malvern Zetasizer Nano ZS) for a 30 minute duration and operating in the backscatter (173°) detection mode. Furthermore, ergodicity of the sample is determined by probing the scattering intensity over a range of positions within the material. For more information on determining ergodicity of a sample using scattering intensity, see our previously published work on dynamic light scattering microrheology (7, 8). The measured correlation function is exported from the instrument and used to derive a mean-squared displacement (MSD) as a function of time windows. The MSD is then Fourier transformed and inputted into the generalized Stokes-Einstein equation to derive the complex modulus of the material. All of the analysis of the exported correlation function is performed using a publicly available code package (found at dlsur.readthedocs.io) as described in previously published methods (7). Furthermore, previously published data shows that the particular probe particles used for DLSμR in this paper exhibits minimal interaction with the HA host–guest material (Figure 6 in (7)).

Small Angle Oscillatory Shear Rheology of HA Host–Guest Materials. The lyophilized HA material is dissolved in 1X PBS (pH 7.4) at the desired concentration and rotated at room temperature overnight. The two components at the desired polymer concentration are mixed together to a final volume of 50μL by pipetting up and down and then transferred to the rheometer stage. Macrorheology was performed using an ARG2 rheometer fitted with a 20 mm, 1° cone geometry. To prevent dehydration, a solvent trap was used for all experiments. Gelation time sweeps were performed at 1 rad/s oscillatory frequency, 1% strain, and 25°C. The equilibrium point was identified by finding the time at which the modulus began to oscillate around a steady value. Following this, frequency sweeps from 0.1 to 100 rad/s were performed at 1% strain and 25°C. Strain sweeps were performed from 0.1% to 10% strain at 1 rad/s oscillatory frequency to ascertain that measurements were performed in the linear regime. For temperature sweeps, the temperature of the stage was set to the desired temperature and, after 10 minutes of equilibration at the new temperature, the frequency sweep as described above was performed at that temperature.

Evaluation of Brachiation Model and Simulated Annealing. The self-consistent calculation using inputs to the model to generate rheological predictions was done computationally using Python. To resolve high frequency rheological predictions, the minimum number of modes (P) included was 500. The script used to generate the rheological predictions is available online through Github from our lab website. Additionally, to obtain fitted parameters to a subset of the experimental data, simulated annealing was performed using a custom Python script with a logarithmic temperature descent

$$T(i) = \frac{T_0}{1 + \alpha \log(1 + i)} \qquad [9]$$

with a starting temperature T_0 of 2000, α of 0.99, and maximum number of iterations of 2000.

Safety Statement. No unexpected or unusually high safety hazards were encountered.

Table S1. 40 kDa Hyaluronic Acid Gel Permeation Chromatography Measurement Results.

Molar mass moments (g/mol)	M_n	$4.395 \times 10^4 (\pm 3.176\%)$
	M_w	$5.965 \times 10^4 (\pm 2.143\%)$
Polydispersity	M_w/M_n	$1.357 (\pm 3.831\%)$

Table S2. Dynamic Light Scattering Measurements of Polystyrene Particles Before and After PEGylation.

	Mean Diameter (nm)
Before PEGylation	466.6
After PEGylation	509.3

Table S3. Sticky Rouse Model Parameters for HA Host–Guest Materials.

Host–Guest wt%	P_0	N	N_s	τ_0	τ_S	M_w	T	Model
CB7–Xyl 5%	1.33×10^{-4}	200	25	4.00×10^{-2}	1.00×10^{-3}	4.00×10^{4}	25	sticky Rouse
CB7–Phe 7.5%	4.67×10^{-4}	200	25	6.50×10^{-2}	1.00×10^{-4}	4.00×10^{4}	25	sticky Rouse
CB7–Ada 3%	3.75×10^{-2}	30	25	4.00	4.72×10^{-10}	4.00×10^{4}	25	sticky Rouse
CB7–Ada 5%	3.75×10^{-3}	30	25	8.00	3.20×10^{-4}	4.00×10^{4}	25	sticky Rouse

Table S4. Brachiation Model Parameters for PDMA Nickel-Histidine Materials. The Brachiation model parameters polymer concentration c (M^{-1}), number of stickers per chain M, number of monomers per chain N, drag coefficient ξ (kg/s for Brach Rouse, Pa·s for Brach Zimm), unbinding rate k_u (s^{-1}), binding rate k_b (M^{-1}s^{-1}), and temperature T ($^\circ$C) were fitted to the 10 wt% rheological data of PDMA Nickel-Histidine networks measured at 4 different temperatures: 5°C, 15°C, 25°C, and 35°C.

Material	c	M	N	ξ	k_u	k_b	T	Model
PDMA-Ni-His-10wt%	0.868	101	5222	2.69×10^{-11}	3.34×10^{-3}	1.78×10^{-6}	5	Brach Rouse
PDMA-Ni-His-10wt%	0.868	101	5222	2.69×10^{-11}	1.05×10^{-2}	5.20×10^{-6}	15	Brach Rouse
PDMA-Ni-His-10wt%	0.868	101	5222	2.69×10^{-11}	2.52×10^{-2}	1.07×10^{-5}	25	Brach Rouse
PDMA-Ni-His-10wt%	0.868	101	5222	2.69×10^{-11}	6.37×10^{-2}	2.46×10^{-5}	35	Brach Rouse

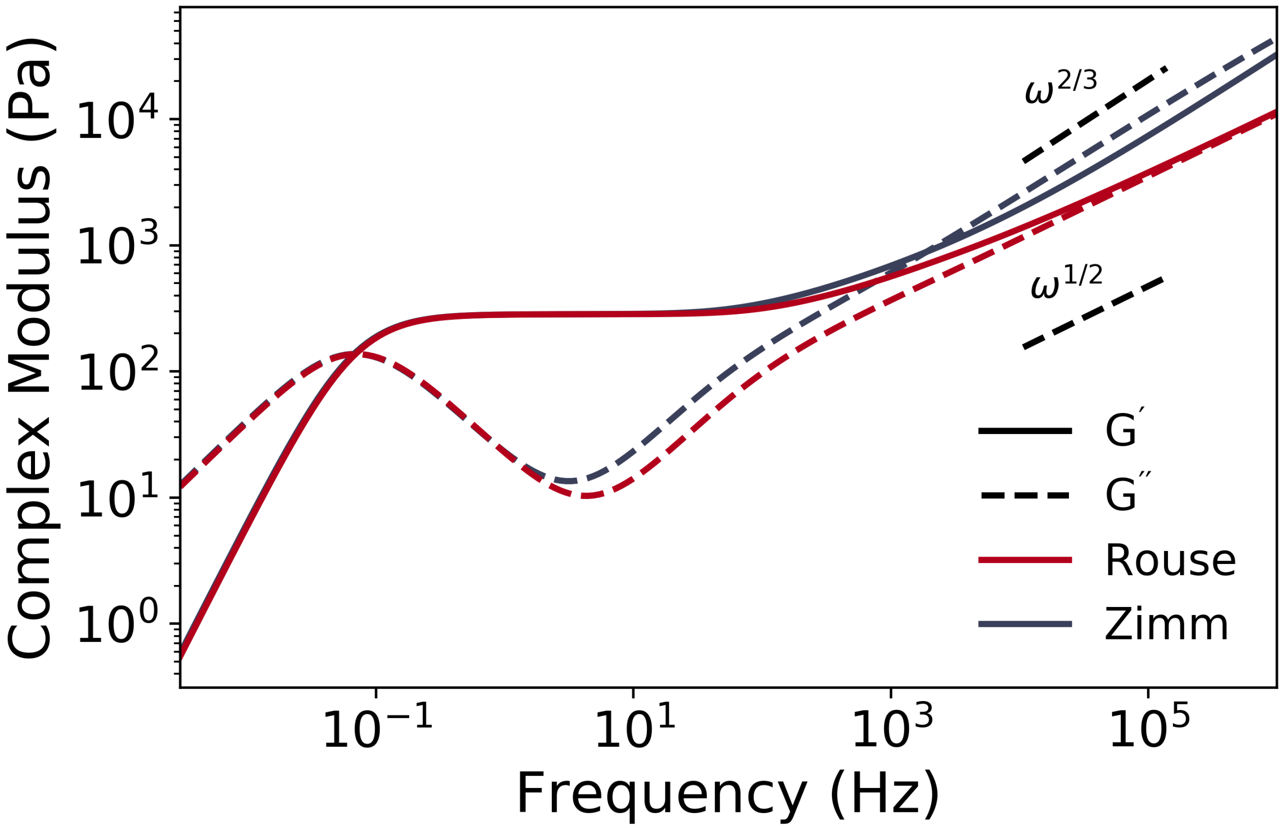

Fig. S1. Modification of the Brachiation model to include hydrodynamic forces. The rheological spectrum as predicted by the Brachiation model is shown here. The red line represents the Brachiation model using the Rouse treatment of the monomer-monomer interactions, while the blue line represents the model using the Zimm treatment. The scaling of each treatment follows what is expected. See Supporting text for details on obtaining the model with the Zimm treatment.

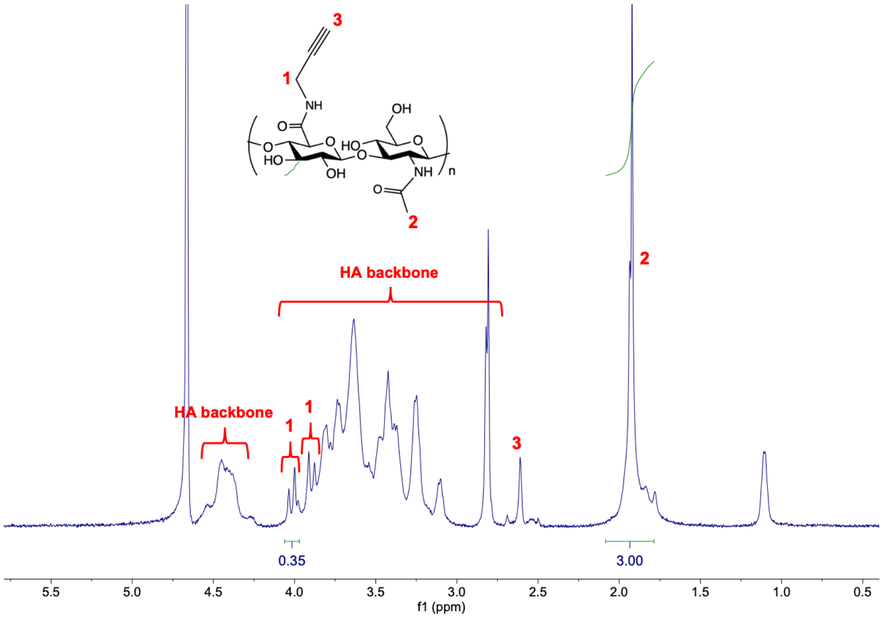

Fig. S2. NMR of HA-Alkyne. NMR of HA-Alkyne shows around a 35% degree of modification of the HA backbone with alkynes.

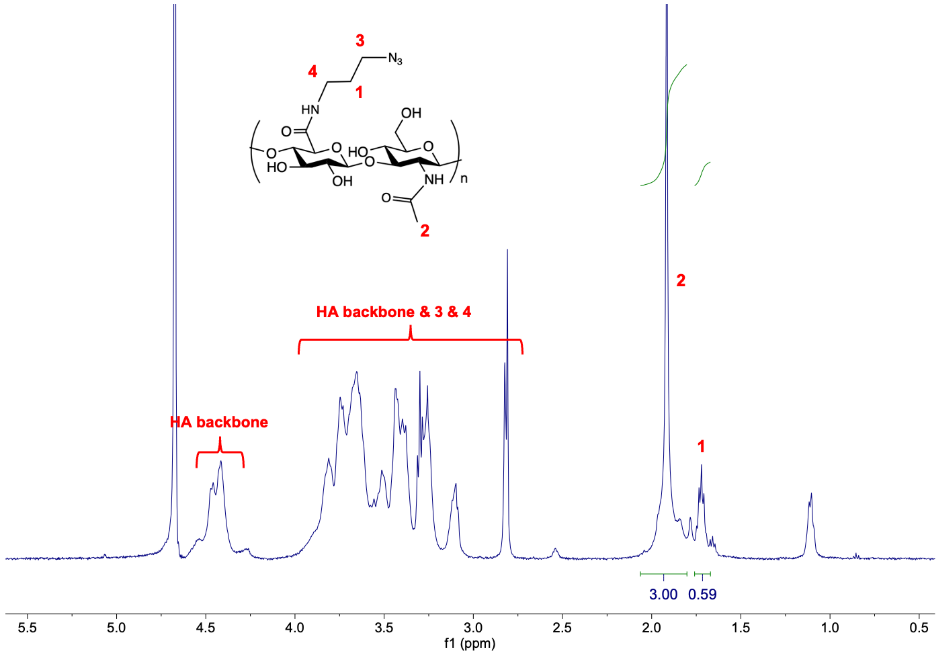

Fig. S3. NMR of HA-Azide. NMR of HA-Azide shows around a 30% degree of modification of the HA backbone with azides.

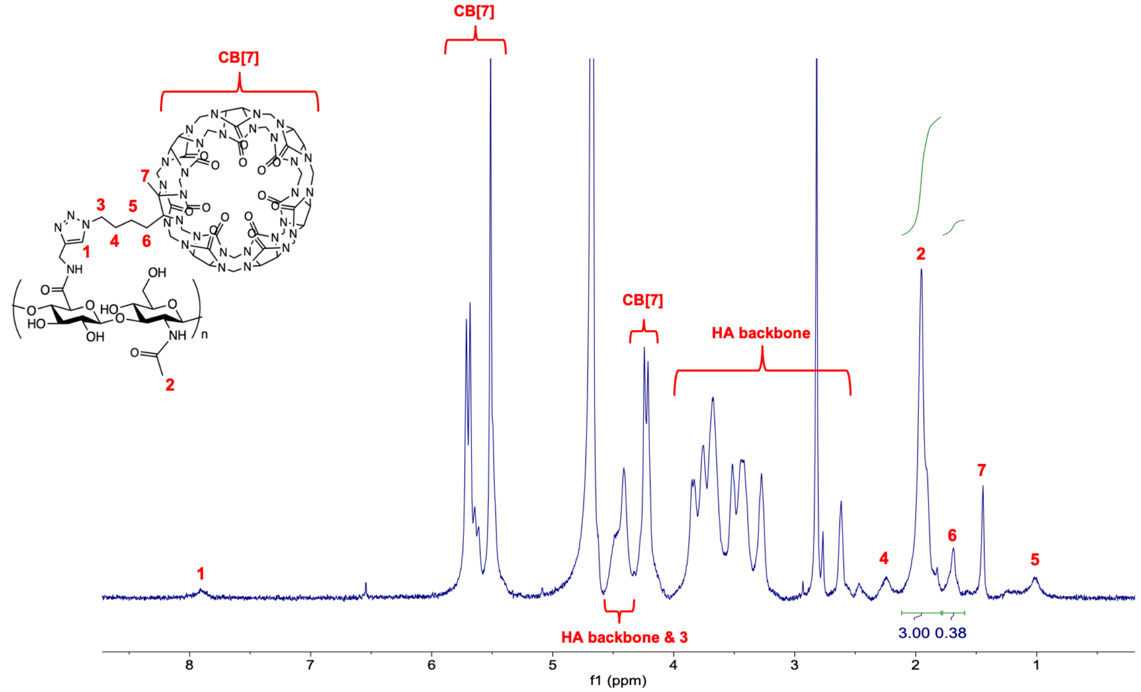

Fig. S4. NMR of HA-CB7. NMR of HA-CB7 shows around a 19% degree of modification of the HA backbone with CB7 groups.

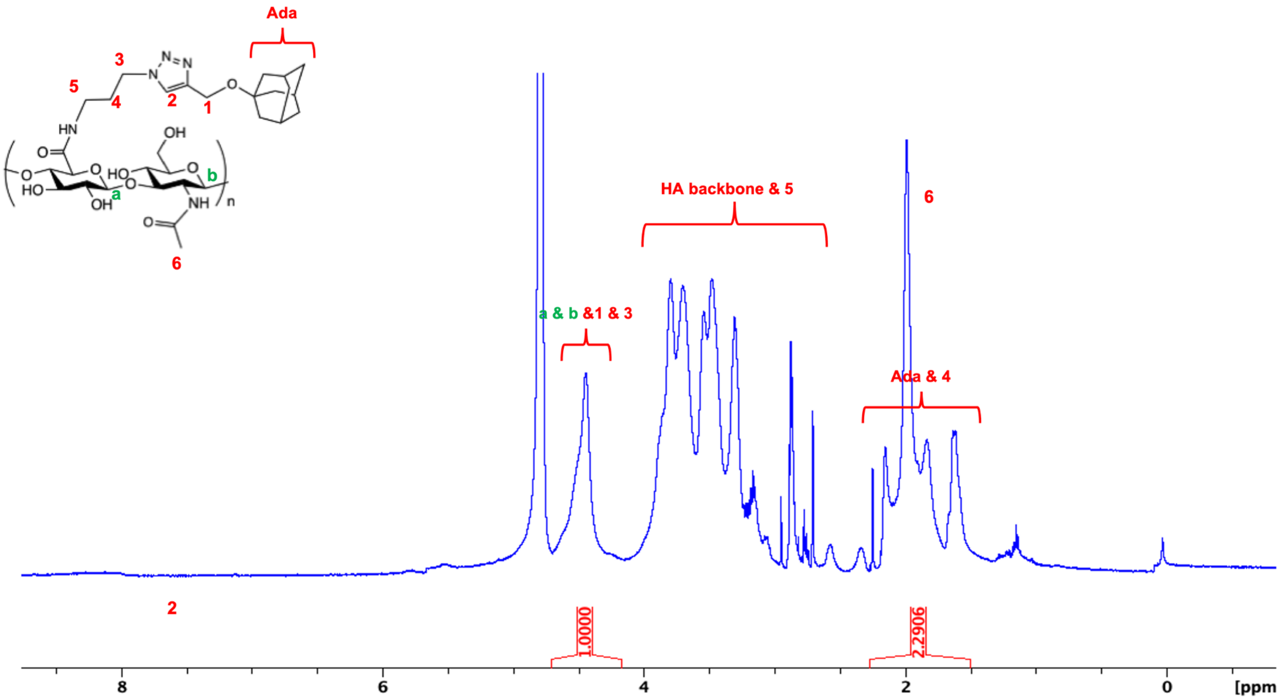

Fig. S5. NMR of HA-Ada. NMR of HA-Ada shows several integrated peaks at around $\delta = 2$ ppm of 2.2906, capturing 6(3H), 4(2H), and Ada(15H). Similarly, the peak around $\delta = 4.5$ ppm is integrated to 1.0 and includes a(1H), b(1H), 1(2H), and 3(2H). We find that the degree of modification is 20.2%.

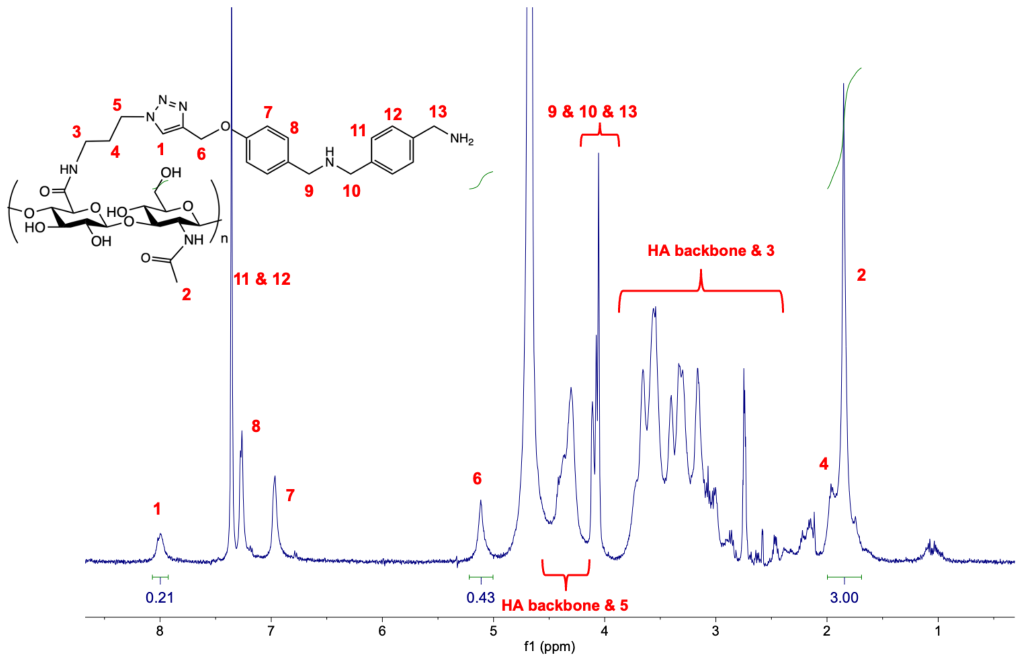

Fig. S6. NMR of HA-Xyl. NMR of HA-Xyl shows around a 21% degree of modification of the HA backbone with Xyl groups.

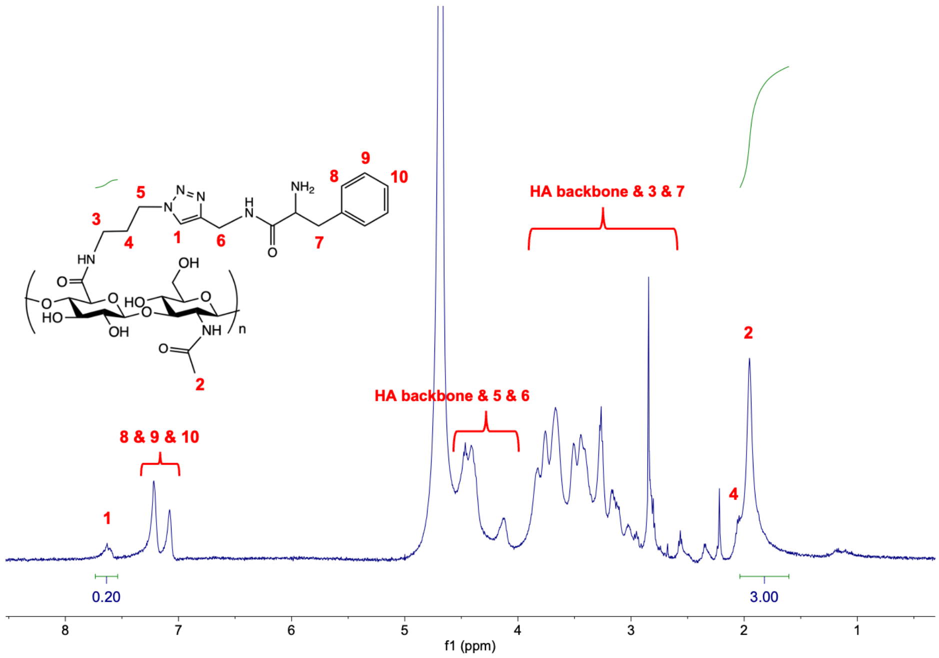

Fig. S7. NMR of HA-Phe. NMR of HA-Phe shows around a 20% degree of modification of the HA backbone with Phe groups.

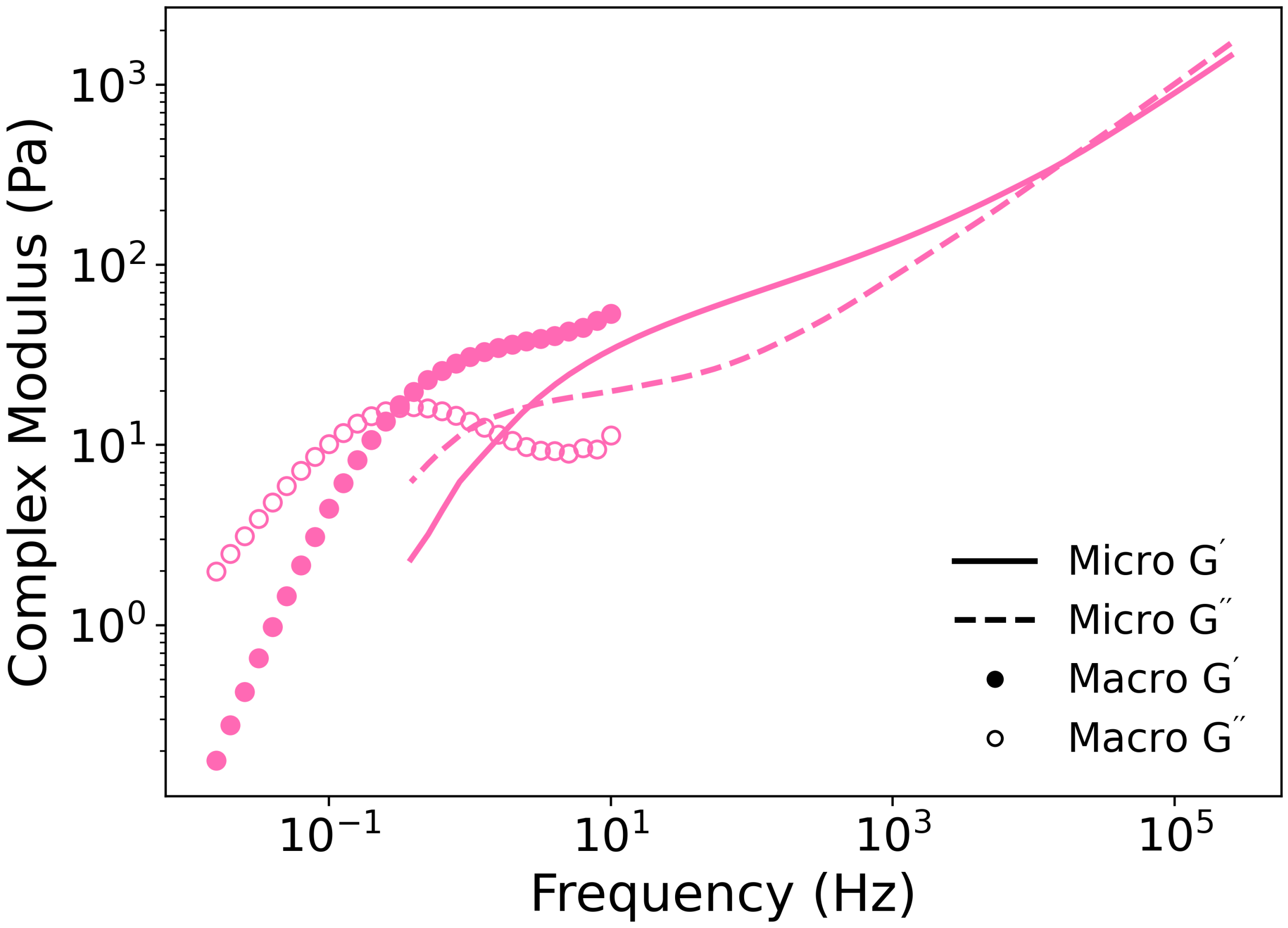

Fig. S8. Macrorheology of HA host–guest gels. The rheological spectra of HA-CB7 and HA-Xyl at 5 wt% are obtained using small angle oscillatory shear rheology. The bulk rheological measurements are compared with rheological data obtained using dynamic light scattering microrheology.

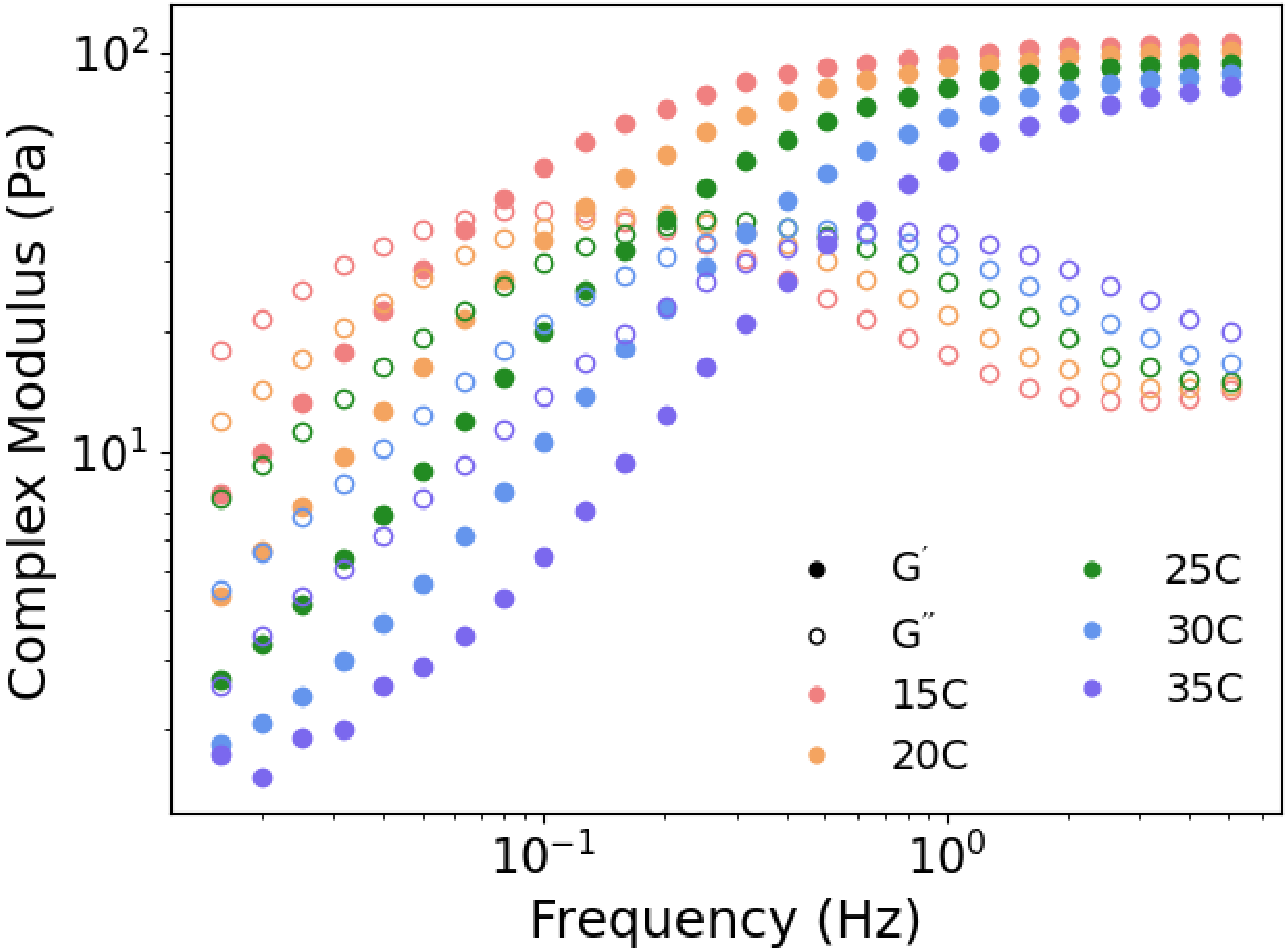

Fig. S9. Temperature Sweep of HA-CB7 and HA-Xyl Gel at 5 wt%. The rheology of a 5 wt% HA-CB7 and HA-Xyl gel was measured using oscillatory shear in the linear viscoelastic regime at temperatures from 15°C to 35°C.

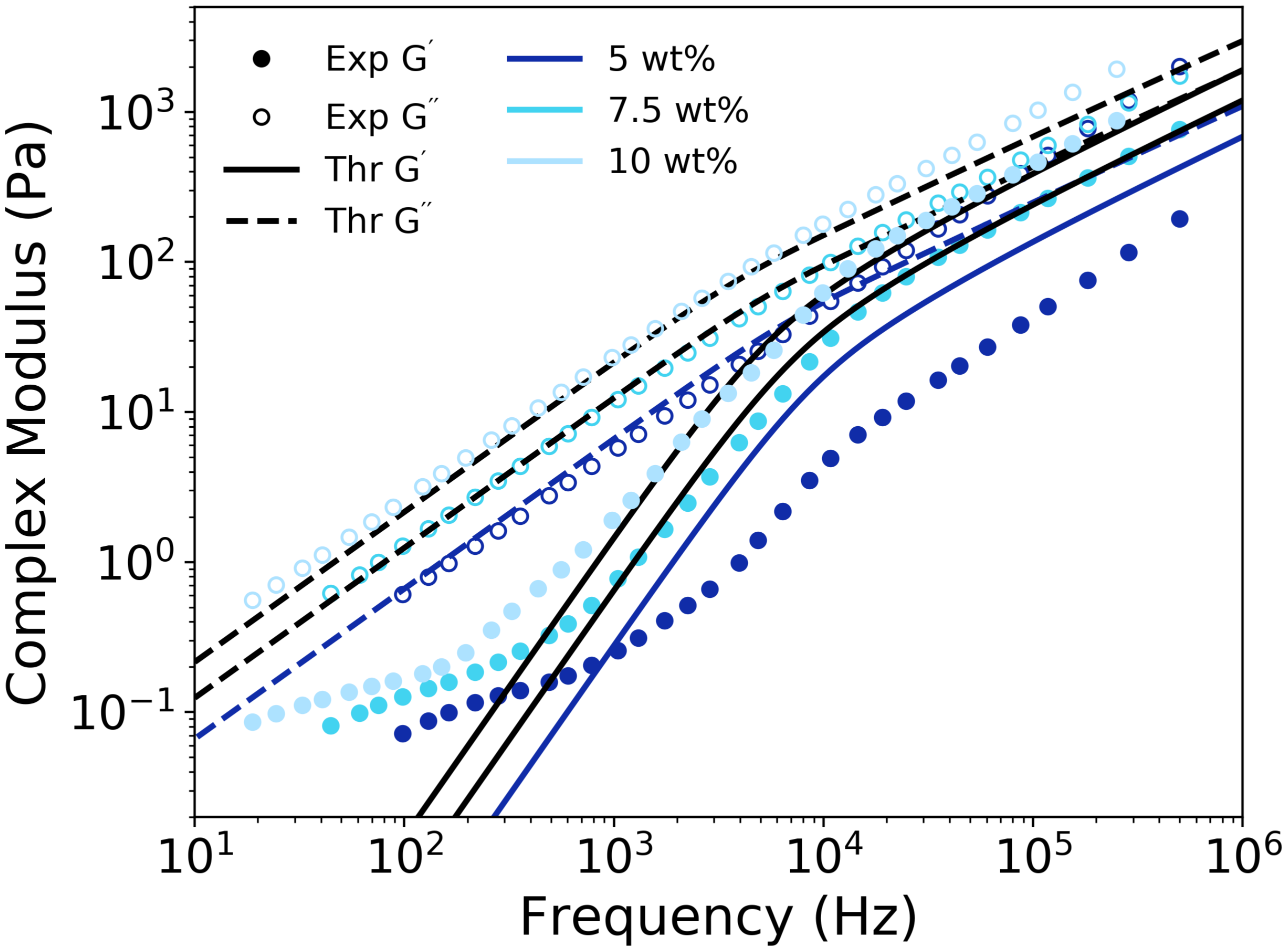

Fig. S10. Brachiation Model Predictions for HA-CB7 and HA-Phe Materials. The rheological spectra of HA-CB7 and HA-Phe are shown at concentrations of 5 wt%, 7.5 wt%, and 10 wt% along with the rheological output from the Brachiation model with parameters from Table 1. The parameters of the Brachiation model were fit to the 5 wt% rheology data and then altered to find the theoretical predictions for the two higher concentrations.

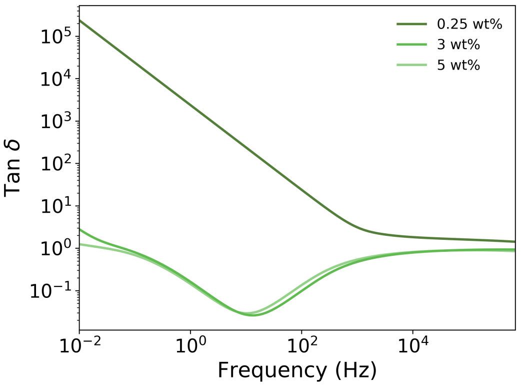

Fig. S11. The tangent δ (=G"/G') for the theoretical predictions of the CB7–Ada host–guest pair materials at all concentrations are plotted to demonstrate the transition from sol to gel phase with emergence of a minimum point in the tangent δ at 3 and 5 wt%.

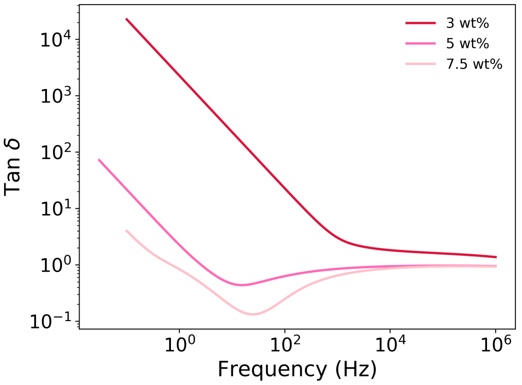

Fig. S12. The tangent δ (=G"/G') for the theoretical predictions of the CB7–Xyl host–guest pair materials at all concentrations are plotted to demonstrate the transition from the sol to the gel phase between concentrations 3 wt% and 5 wt% with the emergence of a minimum point in the tangent δ.

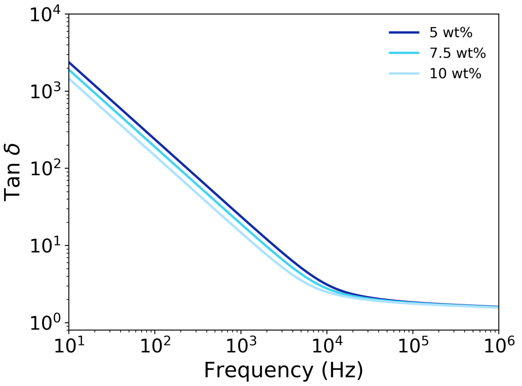

Fig. S13. The tangent δ (=G"/G') for the theoretical predictions of the CB7–Phe host–guest pair materials at all concentrations are plotted to demonstrate that all concentrations are within the sol phase due to the lack of a minimum in the tangent δ.

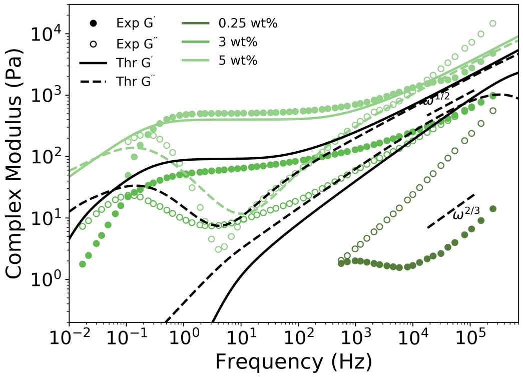

Fig. S14. The Brachiation model predictions for all concentrations of HA-CB7 and HA-Ada if only changing the concentration parameter.

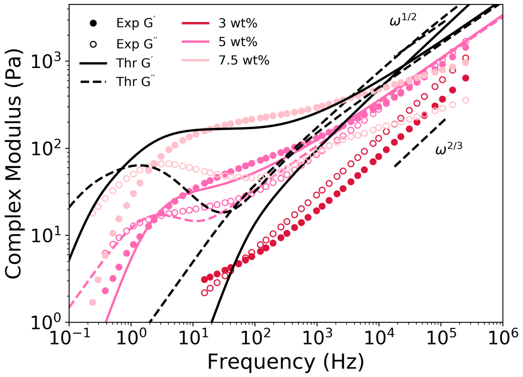

Fig. S15. The Brachiation model predictions for all concentrations of HA-CB7 and HA-Xyl if only changing the concentration parameter.

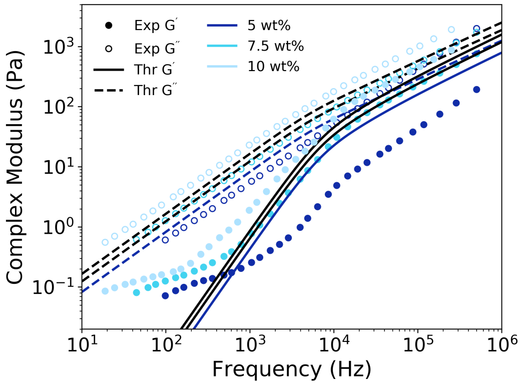

Fig. S16. The Brachiation model predictions for all concentrations of HA-CB7 and HA-Phe if only changing the concentration parameter.

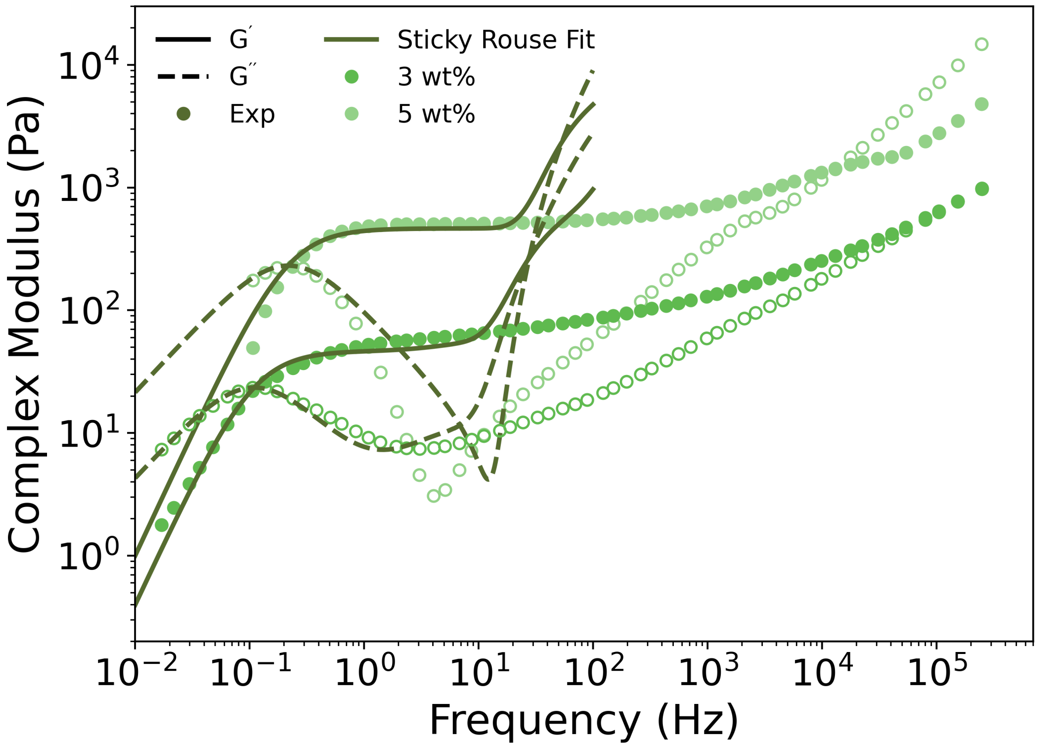

Fig. S17. Plotted sticky Rouse model fits using parameters in Table S3 (dark lines) with experimental rheological data (circles) for 3 wt% and 5 wt% HA-CB7 and HA-Ada networks.

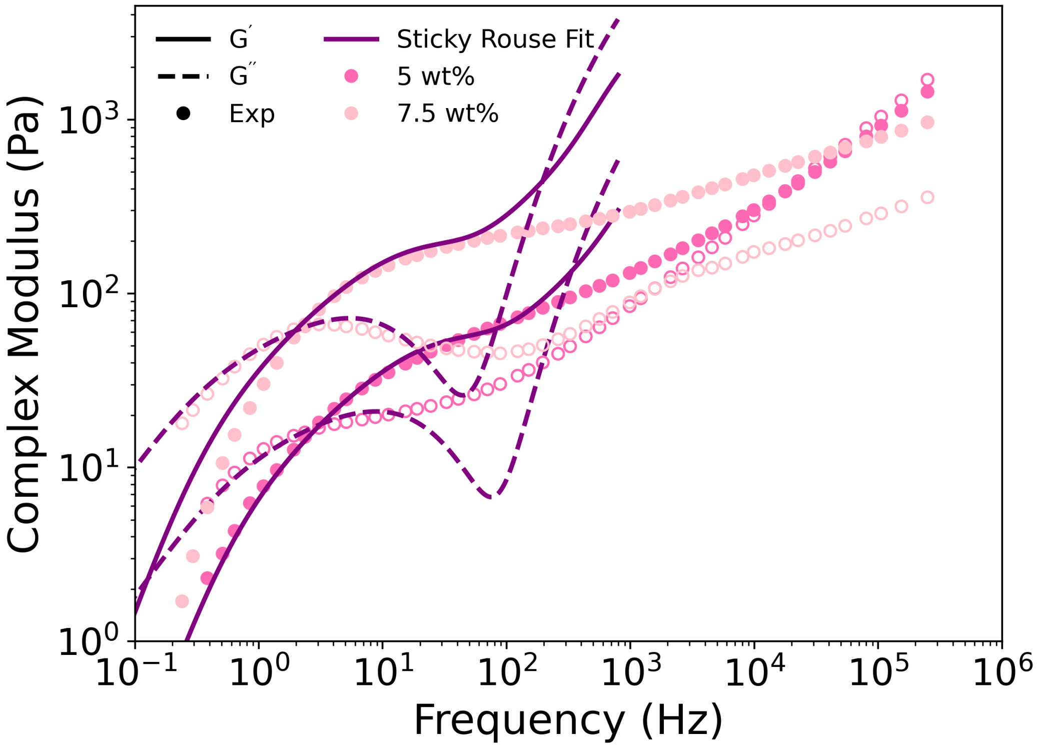

Fig. S18. Plotted sticky Rouse model fits using parameters in Table S3 (dark lines) with experimental rheological data (circles) for 5 wt% and 7.5 wt% HA-CB7 and HA-Xyl networks.

References

1. PC Cai, BA Krajina, AJ Spakowitz, Brachiation of a polymer chain in the presence of a dynamic network. *Phys. Rev. E* **102**, 020501 (2020).
2. M Doi, SF Edwards, *The theory of polymer dynamics.* (Oxford University Press) Vol. 73, (1988).
3. S Tang, BD Olsen, Relaxation processes in supramolecular metallogels based on histidine–nickel coordination bonds. *Macromolecules* **49**, 9163–9175 (2016).
4. C Oelschlaeger, M Cota Pinto Coelho, N Willenbacher, Chain flexibility and dynamics of polysaccharide hyaluronan in entangled solutions: a high frequency rheology and diffusing wave spectroscopy study. *Biomacromolecules* **14**, 3689–3696 (2013).
5. B Vinciguerra, et al., Synthesis and self-assembly processes of monofunctionalized cucurbit [7] uril. *J. Am. Chem. Soc.* **134**, 13133–13140 (2012).
6. L Zou, AS Braegelman, MJ Webber, Dynamic supramolecular hydrogels spanning an unprecedented range of host–guest affinity. *ACS applied materials & interfaces* **11**, 5695–5700 (2019).
7. PC Cai, et al., Dynamic light scattering microrheology for soft and living materials. *Soft Matter* **17**, 1929–1939 (2021).
8. BA Krajina, et al., Dynamic light scattering microrheology reveals multiscale viscoelasticity of polymer gels and precious biological materials. *ACS central science* **3**, 1294–1303 (2017).
